# Arousal, interindividual differences and temporal binding a psychophysiological study

**DOI:** 10.1007/s00426-024-01976-3

**Published:** 2024-05-28

**Authors:** Anna Render, Hedwig Eisenbarth, Matt Oxner, Petra Jansen

**Affiliations:** 1 Faculty of Human Sciences, University of Regensburg, Regensburg, Germany; 2 Victoria University of Wellington, Wellington, New Zealand; 3 University of Passau, Passau, Germany; 4 Wilhelm Wundt Institute for Psychology, University of Leipzig, Leipzig, Germany

**Keywords:** Sense of agency, Temporal binding, Arousal reactivity, Eye blink rates, Sexual arousal

## Abstract

The sense of agency varies as a function of arousal in negative emotional contexts. As yet, it is unknown whether the same is true for positive affect, and how inter-individual characteristics might predict these effects. Temporal binding, an implicit measure of the sense of agency, was measured in 59 participants before and after watching either an emotionally neutral film clip or a positive film clip with high or low arousal. Analyses included participants’ individual differences in subjective affective ratings, physiological arousal (pupillometry, skin conductance, heart rate), striatal dopamine levels via eye blink rates, and psychopathy. Linear mixed models showed that sexual arousal decreased temporal binding whereas calm pleasure had no facilitation effect on binding. Striatal dopamine levels were positively linked whereas subjective and physiological arousal may be negatively associated with binding towards actions. Psychopathic traits reduced the effect of high arousal on binding towards actions. These results provide evidence that individual differences influence the extent to which the temporal binding is affected by high arousing states with positive valence.

**Supplementary Information:**

The online version contains supplementary material available at 10.1007/s00426-024-01976-3.

## Introduction

### Sense of agency

We occasionally face situations where we feel a lack of control over our actions, or we feel disconnected with their consequences. For example, we may say things in the heat of an angry moment, or even break a dish in an outburst. Later we come to regret or even disavow those actions, despite knowing that we acted voluntarily at the time. This feeling of control over the consequences of one’s actions, or of causing external events by voluntary action, is called the sense of agency (Gallagher 2000; Haggard & Tsakiris 2009). The sense of agency is essential to interacting with the environment (Synofzik et al. 2013) and serves central social functions, such as attributing social or legal responsibility (Frith 2014; Haggard 2017; Haggard & Tsakiris 2009; Moore 2016).

It is commonly distinguished between *feelings* of agency, reflecting non-conceptual implicit aspects of the sense of agency, and *judgments* of agency, reflecting its explicit aspects (Synofzik et al. 2008). Over the last two decades, several indirect measures for the sense of agency have been developed (for a review see Moore 2016). A problem facing the sense of agency research today is that these different implicit methods do not seem to capture the same phenomena (Dewey & Knoblich 2014; Siebertz & Jansen 2022), and that explicit agency attributions fail to consistently correspond to implicit measures (Dewey & Knoblich 2014; Obhi & Hall 2011; Reis et al. 2022; Synofzik & Vosgerau 2012). One proxy measure of the implicit sense of agency is temporal binding, which describes a subjective compression of time between a self-initiated action and its perceived outcome (Haggard et al. 2002; Haggard & Tsakiris 2009). Interestingly, not only explicit measures but also implicit measures have been shown to be affected by a retrospective self-serving bias to enhance the self-concept (Christensen et al. 2016; Yoshie & Haggard 2013), attributing positive actions and outcomes to oneself, while distancing oneself from negative actions and outcomes (Christensen et al. 2016).

### Moderating factors for the sense of agency

To investigate this, a body of research has focused on negative action outcomes, such as fearful or angry human vocalisations, which reduce the sense of agency over the committed action compared to either positive or neutral outcomes (Barlas et al. 2018; Christensen et al. 2016; Gentsch et al. 2015; Takahata et al. 2012; Yoshie & Haggard 2013), (Moreton et al. 2017, for contrary findings). It should be noted that results were dependent on predictability and anticipation of action outcome (for a review, Kaiser et al. 2021). From this,Christensen et al. (2019) suggest that a negative emotional state during action performance might cause a reduction in the sense of control over one’s actions and their external outcomes as well.

In the two-dimensional view of affect (Russell & Barrett 1999), each emotional experience can be described by dimensions of valence (positive vs. negative) and arousal (high vs. low). Following this classification, previous research shows that affect with positive valence enhances the sense of agency (Aarts et al. 2012) and that high arousal states with negative valence, such as fear and anger, reduce temporal binding towards actions (Christensen et al. 2019). Results from other studies highlight factors that affect the direction of effects during high arousal: valence, the method of arousal induction, and the temporal binding measure used. For example, temporal binding is increased when arousal is induced by colour and movement of abstract shapes, such as red jumping squares, and when binding is measured with the interval estimation procedure (Wen et al. 2015). In contrast, temporal binding as measured by the Libet clock task is partially reduced when arousal is induced via emotionally ambivalent film clips (Render & Jansen 2020).

To date, studies investigating the relationship between arousal and the sense of agency (Christensen et al. 2019; Wen et al. 2015) have not explored the potential effects of positive valence, nor taken inter-individual differences in subjective and physiological arousal or valence response into account.

A physiological factor often highlighted in clinical groups that is associated with the response to valence or reward is dopaminergic activity (Castrellon et al. 2019). Interestingly, dopaminergic dysfunction has been shown to lead to alterations in temporal binding, seen in psychiatric and neurological disorders such as schizophrenia (Di Plinio et al. 2019; Graham-Schmidt et al. 2016; Moore et al. 2011; Voss et al. 2010) and Parkinson’s disease (Saito et al. 2017). Hints of the dopaminergic system’s involvement in temporal binding have also been found in healthy subjects using ketamine as a model for psychosis (Moore et al. 2011) and when primed with positive, low-arousal pictures via a positive relationship between temporal binding and high baseline blink rates (Aarts et al. 2012).

Spontaneous eye blink rates are a non-invasive indirect marker of striatal dopamine (Jongkees & Colzato 2016; Sadibolova et al. 2022), which can predict hypo- and hyperdopaminergic activity, with higher blink rates predicting higher dopaminergic function (Jongkees & Colzato 2016). It should be noted that the relationship between dopamine and blink rates is complex; when given a dopamine agonist, eye blink rates *increase* in individuals with low baseline blink rates but *decrease* in individuals with high baseline blink rates. This indicates that baseline eye blink rates, and presumably the associated dopamine level, modulate the effect of dopamine manipulations on blinking (Cavanagh et al. 2014). Similar reductions in blink rates have been observed for sexual interest (Hecker et al. 2009), as well as during positive, high arousal affect (*sexual arousal*) induced by erotic imagery (Maffei & Angrilli 2019). In temporal binding research, participants with high baseline blink rates showed increased temporal binding when primed with positive, low-arousal pictures (Aarts et al. 2012). Nevertheless, it is still unknown whether the temporal binding in individuals with higher striatal dopamine levels is also increased when positive valence is induced at high arousal levels.

A reliable way to assess physiological arousal is to use pupil dilation, skin conductance, and heart rate. The psychosensory pupil response is pupil dilation through stimulation such as increased sexual arousal or mental effort (e.g., Beatty & Lucero-Wagoner 2000). Similarly, skin conductance varies directly with reports of arousal, independent of whether the experience is reported as pleasant or unpleasant (for discussion see, Lang et al. 1993). Given the different aspects of arousal reflected in these measures, combining these physiological correlates with self-report on a cognitive level could allow a holistic moment-by-moment reflection of participants’ arousal states on temporal binding.

A personality trait that is particularly relevant to examining alterations in arousal and valence reactivity is psychopathy. On a behavioural level, high levels of psychopathy are associated with reduced emotional responding and empathy, callousness, deceitfulness, and impulsive, irresponsible, and antisocial behaviour (Hare & Neumann 2008). On a physiological level, psychopathy has been widely discussed to be related to differences in neural functionality such as hypersensitivity in the ventral striatum or nucleus accumbens dopamine release during both reward and loss processing (Buckholtz et al. 2010; Murray et al. 2018). There are also psychopathy-associated variations in the physiological responses to arousal through alterations in the autonomic nervous system (for a meta-analysis see Looff et al. 2022). In fact, a well-replicated biological marker of psychopathy is low resting heart rate (Looff et al. 2022; Portnoy & Farrington 2015), and a reliable estimate of psychopathy is low skin conductance response reactivity (Looff et al. 2022). With respect to the mechanisms leading to reduced affective or arousal response in psychopathy, traditional theories of psychopathy either claim deficits in emotional and/or cognitive processes, i.e. reductions in emotional experience/reactivity and the inability to adaptively allocate processing resources such as attention (see for a review, Groat & Shane 2020). While there is a large body of research confirming aberrant emotional/ cognitive processing in individuals high on psychopathy, evidence that these alterations are due to inabilities is absent (Shane & Groat 2018). In fact, individuals high on psychopathy do not show alterations in emotional experiences or cognitive processing under certain specifically designed contexts such as when incentives are given (Newman et al. 2010; Shane & Groat 2018). Thus, more recently, a motivational framework has been suggested (Groat & Shane 2020) assuming that individuals high on psychopathy can upregulate their emotional responses given sufficient motivation. If psychopathy is associated with a reduced emotional responding and a reduced autonomic nervous system response, highly arousing states may have reduced effects on temporal binding in individuals high on psychopathy. At the same time, if psychopathy is linked to a hypersensitive reward system, incentivised pleasant states may intensify the effects on temporal binding.

To induce positive affect that differs in arousal intensity, previous studies have used short film clips showing pleasant social interactions (low arousal) or pornographic material (high arousal) (e.g., Gabert-Quillen et al. 2015). Highly arousing positive states, such as sexual arousal, impact other cognitive, perceptual, and behavioural processes. These include the ability to predict one’s judgments and behaviour (Ariely & Loewenstein 2006), decision-making processes (Ditto et al. 2006), and self-control and self-restraint (Skakoon-Sparling & Cramer 2016). In contrast, positive affect that is low on the arousal spectrum (which we refer to as *calm pleasure*) facilitates cognitive processes; for example, it promotes creative problem-solving, improves cognitive flexibility (Chiew & Braver 2011), and increases awareness of intention to act (Rigoni et al. 2015). The effects of positive affect on the sense of agency will be measured via temporal binding.

### Measuring sense of agency with temporal binding

As mentioned earlier, the implicit measure of temporal binding, also referred to as intentional binding (Haggard et al. 2002), refers to a subjective compression of the time between a self-initiated action (e.g., key press) and its perceived outcome (e.g., a tone) (Haggard et al. 2002). This is indicated by a forward shift in the estimated time of an action toward its outcome (action binding) and the backward shift of the perceived time of an outcome toward a causal action (outcome binding) (Lush et al. 2019). The temporal binding effect occurs whenever cause and effect are linked, and even when agency and intentionality are controlled (Buehner & Humphreys 2009; Kirsch et al. 2019). In more recent years, a body of research has indicated that temporal binding is based on causation rather than intentional action (e.g., Buehner 2012). This suggests that other factors such as predictability, attentional processes, or perceptual certainty underpin temporal binding (Hon 2023; Klaffehn et al. 2021).

### Subcomponents of temporal binding

Action and outcome binding, the two subcomponents of temporal binding, are thought to be based on different characteristics for the two perceptual shifts (for meta-analysis see Tanaka et al. 2019) and have been shown to be uncorrelated (Siebertz & Jansen 2022; Tonn et al. 2021).

Action shifts rely on whether one can control outcome onsets with voluntary actions. Thus, action shift occurs when activating learned action–outcome associations by planning and executing actions (Moore & Haggard 2008). Action binding is thus thought to capture a specific impairment in action planning or generating an action outcome prediction reflecting the deficits in the dopaminergic system involved in action execution (Tanaka et al. 2019).

In contrast, outcome shifts rely on the degree to which participants can predict the action outcome onset. Therefore, outcome shift occurs based on comparing predicted and observed outcomes (Tanaka et al. 2019). Consequently, outcome binding may depend more on physical characteristics of the outcome due to its direct association with sensorimotor prediction (Christensen et al. 2019; Wolpe & Rowe 2014).

From this, it has also been argued that action binding may be the more informative measure of action-outcome association than outcome binding, as the former is independent of the physical characteristics of the outcome event (Christensen et al. 2019). Christensen suggests that a large positive correlation between outcome bindings in the active and passive condition indicates that outcome binding results from mechanisms that are part of action perception, regardless of whether the agent is the self or another. This concern does not apply to action binding. Following this notion, we investigated effects on action and outcome binding separately focusing our hypotheses on action binding only.

### Research gap and hypotheses

Although the influence of negative affect on temporal binding has been investigated (Christensen et al. 2019), the relationship between positive affect, inter-individual differences in affective response, and temporal binding has not yet been clarified. In this study, we addressed this gap, first investigating how positive affect differing in arousal intensity influences each binding component. We predicted that action binding would be reduced after sexual arousal and increased after calm pleasure. We did not have any specific hypothesis for outcome binding but conducted exploratory analyses for those effects.

Second, to model inter-individual differences in subjective and physiological arousal and valence response we included subjective affect ratings, pupil dilation and changes in striatal dopamine levels via eye blink rates (in response to a stimulus) in our analysis. During sexual arousal, large increases in pupil dilation were expected to correlate with a decrease in action binding, while during calm pleasure, moderate increases in pupil dilation were anticipated to correspond to an increase in action binding. For striatal dopamine levels, we expected that action binding increases as eye blink rates increase, whereas action binding decreases as eye blink rates decrease dependent on baseline activity. Specifically, we anticipated higher blink rates in individuals with low baseline activity and lower blink rates in those with moderate and high baseline activity. Exploratory analyses tested whether striatal dopamine levels mirror the extent of arousal and valence induced via positive film clips by reducing or intensifying these effects.

Third, to investigate the role of inter-individual differences in the affective response from a personality perspective, we included psychopathic traits in our model. For participants high on psychopathy, we hypothesized increased action binding. This is because preliminary evidence suggests that individuals high on psychopathy interpret their own actions correctly (Brazil et al. 2011). Given that higher levels of psychopathy are associated with a reduced autonomous nervous system response and a hyperactive reward system, it was hypothesized that psychopathy may attenuate the effects of sexual arousal induction while increasing the effects of calm pleasure. These hypotheses were tested in exploratory analyses.

## Methods

This study was preregistered on Open Science Framework (OSF) at the start of data collection (osf.io/pskmh, anonymous link for peer review: https://osf.io/z7spx/?view_only=f30d881f55114285b48972996593b970). The preregistration adheres to the disclosure requirements of OSF. All data, materials, and code used are available on OSF. The pre-registration includes additional hypotheses that are discussed in the Supplementary Materials, section ‘Methods and Results for Additional Hypotheses in Pre-registration’.

### Participants

The required sample size was estimated using the program G by power (Faul et al. 2009). Previous experiments with similar designs showed a large range of effect sizes for temporal binding (η_p_^2^ = 0.14 (Aarts et al. 2012), η_p_^2^ = 0.19 (Christensen et al. 2019), η_p_^2^ = 0.31–0.40 (Di Costa et al. 2018)). Using the effect size in the mid-range and from the most comparable experimental design (η_p_^2^ = 0.19), we performed a power analysis for repeated measures analysis (within-between interaction, three groups, two measurements). With a power of 0.9, an α = 0.05 and a non-sphericity correction of 1, a sample size of 60 was estimated to detect the effect. Since a higher power was found for mixed models, the determined sample size should be sufficient (Hilbert et al. 2019).

We recruited participants for the study via flyers and social media platforms. Due to COVID-19-related interruption, testing was stopped at 59 instead of 60. Inclusion criteria were an age of between 18 and 35 years (as dopamine transporters decrease with age (Volkow et al. 1996) and no use of glasses (which could cause glare issues in the eye tracker). The 59 participants were pseudo-randomized (considering gender balance) into one of three groups with different affect inductions. Due to human error, one participant was assigned to the wrong group (sexual arousal *N* = 21, neutral control *N* = 20, calm pleasure *N* = 18); gender balance was met for the sexual arousal and the control group, while the pleasure group had a higher proportion of females. Analyses were performed on de-identified data. Participants provided information about their age (range: 18 to 35 years, *M* = 23.75, *SD* = 4.21) and gender (26 participants identified as male, 31 as female, 2 as non-binary).

Ethical approval for the study was obtained from the School of Psychology Human Ethics Committee, by delegated authority of the Victoria University of Wellington Human Ethics Committee (#028117), prior to the commencement of any testing activities. Participants were informed about the purpose of the study, gave their written consent prior to participation and received vouchers as compensation.

### Materials

#### Apparatus and stimuli

The experiment was run on an Acer PC with a 22-in. flat-screen monitor with 1024 × 768 px resolution and a 120-Hz refresh rate. The viewing distance was maintained by a chin rest 60 cm from the screen. The experiment was programmed and run in MATLAB (The MathWorks Inc. 2018).

#### Physiological measures

Physiological measures included pupil dilation, eye blink rates, skin conductance and heart rate. Data was collected and analysed separately for the temporal binding tasks and for the film clips.

##### Pupillometry and eye blink rates

The area of the right pupil (in arbitrary units) was recorded using an EyeLink 1000-Plus desktop mounted eye-tracker (SR Research Ltd., Mississauga, ON) using a 250-Hz sampling rate. Participants were tested in a dimly lit room. Two nine-point calibrations and validations of the eye tracker were performed at the beginning of the session. Before each trial, a manual drift check was performed, and if necessary, the calibration and validation were performed again.

Pupil data were pre-processed with the gazer package (Geller 2019) in R. Pupil data were de-blinked (blinks identified, removed and interpolated during the blink period and across a longer segment that extends before and after the blink). Blinks and a 200ms window around each were identified by EyeLink algorithms and eliminated as generally recommended. Time windows around blinks were extended with interpolation starting 100-200ms before and after the blink (Nyström et al. 2016), thereby eliminating spurious samples caused by the closing and opening of the eyelids. Missing data of blinks and pupil size were reconstructed using linear interpolation (Bradley et al. 2008). Smoothing was done with 5-point moving average. Subtractive baseline correction was conducted to control for variability in overall pupil size arising from non-task related (tonic) state of arousal (Zekveld et al. 2018). Specifically, the median pupil size during the baseline period 0 to 100ms after each trial onset was calculated (Mathôt et al. 2018). For the film clips, we used the first 500ms from film clip onset as baseline and divided the rest of the film clip into three equally long intervals.

For subjects and items whose amount of missing data was above the threshold of 20% (Winn et al. 2018), artefact rejection was performed. A second pass was performed in addition to interpolation to guarantee that the data was not still contaminated by rapid pupil size disturbances with a median absolute deviation (Kret & Sjak-Shie 2019). Lastly, data were down sampled and aggregated per trial.

For spontaneous eye blink rates, blinks identified by EyeLink were used. Rates per minute within a trial were calculated.

##### Skin conductance

Skin conductance was collected and analysed following established guidelines (Boucsein 2012) via ADInstrument bioamps and converted from analogue to digital signals by ADInstruments Powerlab 16/30 and then recorded in LabChart 8.0.1. Skin conductance was recorded from the medial phalanges of the index and ring fingers using ADInstruments bipolar dry stainless steel GSR electrodes (MLT116F) and a ML116 AC GSR Amp with a sampling rate of 1 kHz. Responses were measured in microsiemens (µS). Skin conductance was operationalized as the peak activity during each trial (maximum values) relative to average baseline activity measured across the 500ms prior to trial onset for each individual. For the film clips, three equally long intervals were used and the peak activity during each interval (maximum values) relative to the average activity before was used. For the first interval, we subtracted the average activity during the first 500ms from film clip onset. For the second interval, we subtracted the mean activity during the first interval. For the third interval, we subtracted the mean activity during the second interval. Thus, individual differences in physiological activity were eliminated through a baseline subtraction. Ceiling effects (out of range) were removed, resulting in 4.45 percent of trials being excluded.

##### Heart rate

Heart rate was collected and analysed following established guidelines (Jennings et al. 1981) via ADInstrument bioamps and converted from analogue to digital signals by ADInstruments Powerlab 16/30 and then recorded in LabChart 8.0.1. Heart rate was measured via electrocardiography (ECG) using disposable adhesive silver/silver chloride (Ag/AgCl) ECG electrodes placed underneath the right collarbone and lower left ribcage, referenced to the left side underneath the collarbone. The ECG signal was amplified by ADInstruments ML138 Octal Bio Amp, sampled at 1 kHz, and band-pass filtered offline between 1 Hz and 400 Hz. Heart rate was calculated by subtracting the mean of the baseline (interval 500ms prior to each trial onset) from the mean value of each trial for each individual. For the film clips, three equally long intervals were used and the mean activity during each interval relative to average activity before each interval. For the first interval, we used first 500ms from film clip onset. For the second interval, we used the mean activity during the first interval. For the third interval, we used the mean activity during the second interval. Thus, individual differences in heart rate were eliminated through baseline subtraction. Less than 40 beats per minute and more than 150 beats per minute were defined as artefacts for heart rate resulting in 3.65 percent of trials being excluded. Heart rate data for two participants were excluded due to technical failure.

#### Film clips

All film clips were pre-tested in an online survey using the online survey platform Qualtrics (see Supplementary Materials) to ensure that the scenes elicited the desired affective response. The sexually arousing film clip showed a threesome scene from the film *Love* (2015) by Gaspar Noé. The calm pleasant film clip showed the opening and closing scenes of the film *Pride and Prejudice* (2005) by Joe Wright, showing aesthetic scenery and a romantic social interaction. The control film clip was naturalistic footage of a pedestrian street scene. Each film clip was about 6.5 minutes long; dialogue was kept to a minimum. Background music was a guitar solo in the sexually arousing film clip and classical music in the pleasant film clip, while the control film clip did not have any background music.

#### Measures

##### Psychopathy assessment

The Psychopathic Personality Inventory-Revised 40 (PPI-R-40) (Eisenbarth et al. 2015), an abbreviated measure (using a genetic algorithm) for the PPI (Lilienfeld & Andrews 1996) and PPI-R (154 Items) (Lilienfeld & Widows 2005), was used to measure psychopathy. The 40 items of the PPI-R-40 load on the subscales cold-heartedness, fearless dominance, and self-centred impulsivity. Responses were given on a four-point scale (false, mostly false, mostly true, true). To screen for manipulative tendencies, two validity scales were included: virtuous responding and deviant responding (Kelley et al. 2016). The PPI-R-40 shows good reliability generally (Cronbach’s α = .8) and in this sample (McDonald’s ω_t_ = .82), demonstrates appropriate convergence with the full-length version (*r*s > .75), and the scales also show comparable criterion validity coefficients for measures of personality and externalizing behaviour (Eisenbarth et al. 2015; Ruchensky et al. 2017).

#### Affective grid

The affective grid (Russell et al. 1989) was used to report subjective affect. The scale has two dimensions, valence, and arousal, ranging from 1 to 9. Participants give one report on a two-dimensional grid to describe their current feelings on each dimension simultaneously.

#### Temporal binding task

To assess temporal binding, the Libet Clock Task (Haggard et al. 2002) was applied as a guiding procedure following previous experimental design descriptions (Aarts & van den Bos 2011). Participants watched an analogue clock, marked with numbers in intervals of 5 (0, 5, 10, 15, 20, 25, …, 55). The duration of one clock rotation was 2560ms; the diameter of the clock face and the arc spanned by the clock hand was 16.56 degrees of visual angle. In each trial, a dot appeared at a pseudo-randomized position and began revolving around the clock. The purpose of the first rotation was for the participants to become used to the speed of the clock. The participants only observed the clock hand rotating in this lap and did not press keys, nor hear tones. The condition-specific events, key presses and/or tones, occurred in the second lap of each trial (Garaizar et al. 2016). Participants’ time estimations of the events were given after the second lap by clicking on the clock face with the cursor (the clock was still visible). If participants failed to press within the second lap, an error message appeared in red in the centre of the screen, which said: “You forgot to press!”, along with an error sound. That screen remained for three seconds, prolonging the trial. This way, the experiment took slightly longer if participants did not respond, i.e., there was a time disadvantage in not responding to increase the motivation to engage in the task. Four trial types were presented in separate blocks that were counterbalanced between participants. In agency trials, pressing a key triggered a tone (100ms, 1000Hz) after a 250-ms delay. In the agency action trials, participants reported their time estimation of the key press. In agency outcome trials, participants reported their time estimation of the tone. In baseline-action trials, there was a key press, but no tone was triggered; afterwards, the time estimation of the key press was reported. In baseline-outcome trials, no key was pressed; the tone was triggered randomly in the second lap and the time estimation of the tone was reported. The clock continued to rotate for a random period between 1500 and 2500ms after the target event (key press or tone).

Participants were asked to look at a fixation cross in the centre of the clock. During trials with key presses, participants were asked to wait for at least one revolution of the dot before pressing the button at a time of their choosing, not to plan, and not to aim for a particular point on the clock (e.g., always at the same time or only at the interval marks of 5). In addition, the instruction asked that they should be as precise as possible (in intervals of 1). Each block contained 25 trials (Moore et al. 2010) and three additional practice trials. The interval of 250ms was chosen as it has been shown to evoke a robust binding effect (e.g., Ruess et al. 2017), thus increasing the confidence that any impairments in the binding effects were brought about by the affect inductions.

### Procedure

The study was part of a more extensive study including several personality traits (see pre-registration and Supplementary Materials for results) and took place at Victoria University of Wellington. The duration for each participant lasted between 1.5-2 hours.

**Fig. 1:**
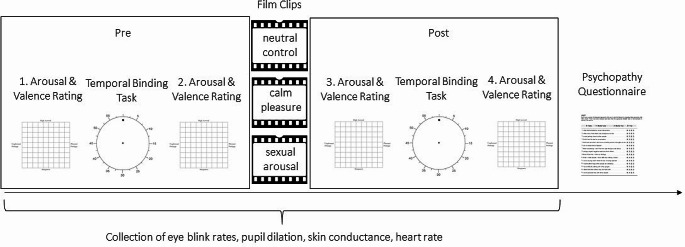
Procedure of the experiment. *Note*: *Participants performed two temporal binding tasks and rated their current feelings on the affective grid. In total, there were four ratings on the affective grid, one before each binding task and one after each binding task, in the following referred to as ratings one to four. In between the temporal binding tasks, participants were pseudo-randomly assigned to watch one of three film clips (sexual arousal, calm pleasure, neutral control). Afterwards, participants completed questionnaires.*

After providing informed consent, participants’ baseline heart rate and skin conductance were recorded, and eye-tracking calibration was performed. Sessions started with a three-minute baseline measure of blink rates (Abusharha 2017; Maffei & Angrilli 2018). Participants rated their subjective feelings on the affective grid four times, before and after each temporal binding task. Each binding task took around 25 minutes. Between the first temporal binding task (pre-induction) and the second temporal binding task (post-induction), participants took a short break (between 5 and 10 minutes). Then participants completed another eye-tracking calibration before watching one of the three film clips, each 6.5 minutes long. To ensure that the affect inductions were maintained after watching the film clips, still images (screenshots) of the film clips were presented between trials for 500ms in the second temporal binding task. In a previous study, we compared two groups with and without emotionally neutral inter-trial images and found that neutral images did not elicit differences in temporal binding (Render & Jansen 2020). Thus, any effects in temporal binding post-induction can be assumed to be due to the content of the intertrial images, and not due to their mere presence. Questionnaires were completed online after the computer-based tasks using the online survey platform SoSci Survey. The duration for completing the questionnaires was 15–20 minutes on average. The entire experimental procedure is illustrated in Fig. 1.

### Statistical analyses

#### Temporal binding

To calculate temporal binding, actual time was subtracted from the reported time in each trial to determine the perception error. Action binding was calculated by subtracting the median error of reported keypress time in the baseline action block from the median error in the agency action block; outcome binding was calculated by subtracting the median error of reported tone time in the agency outcome block from the median error in the baseline outcome block (Moore et al. 2010; Moore et al. 2011). As there was a limited number of trials in each block, medians rather than means were used to eliminate outliers (Pockett & Miller 2007). A higher binding score refers to a smaller interval in perception between key press and tone. One participant reported that they mixed up two blocks of the temporal binding experiment, so the corresponding action binding score was not used in the analysis.

To calculate internal consistency, McDonald’s Omega (ω_t_) was used. Omega is a model-based and factor analytic coefficient used to estimate reliability (true variance relative to observed variance). Its value is influenced by all modelled sources of common variance. As a reliability coefficient, the .70 benchmark for acceptability was used (Rodriguez et al. 2016). For the temporal binding pre-induction measurement, good McDonald’s ω_t_ values of .83 for baseline action, .80 for baseline outcome, .76 for agency action, and .84 for agency outcome were obtained.

For test-retest reliability, the intraclass correlation coefficient (ICC) estimates and their 95% confidence intervals were calculated for the control group (*N* = 20) based on a single-rating, absolute-agreement, 2-way mixed-effects model. The model shows ICC = 0.88 for baseline action (*95% CI = [*0.73, 0.95]), ICC = 0.85 for baseline outcome (*95% CI = [*0.67, 0.94]), ICC = 0.57 for agency action (*95% CI = [*0.19, 0.80]) and ICC = 0.79 for agency outcome (*95% CI = [*0.54, 0.91]). According to the interpretation guidelines of Koo and Li (2016), the ICC value for agency action would be interpreted as moderate and the baseline action, baseline outcome, and agency outcome values would be interpreted as good.

#### Manipulation check

Subjective affect was tested with mixed measures ANOVAs with between factor group (sexual arousal, calm pleasure, neutral control) and within factor time (pre- and post-film clips, the second and third rating of the four total ratings). Dependent factors were arousal and valence scores from the ratings on the affective grid.

To assess physical arousal during the film clips, we ran mixed measures ANOVAs with between factor group (sexual arousal, calm pleasure, neutral control) and within factor interval (film clip baseline, beginning, middle and end interval of the film clips). As the film clip baseline, we used the 500ms from the movie onset. The film clips were split into three equally long intervals because they were expected to differ in their arousal intensity. The sexually arousing clip opened with the sexual engagement scene in the first interval, but the calm pleasure film clip showed the social interaction scene in the second interval. Dependent factors were pupil dilation, skin conductance, and heart rate. Post-hoc tests were performed with the ‘emmeans’ package (Searle et al. 1980). Effect sizes were reported as partial eta squared (η_p_²), where .01 is considered a small effect size, .06 medium effect and .14 a large effect (Cohen 1988). Confidence intervals for partial eta squared are fixed at the upper bound.

To test whether psychopathy is associated with alterations in the arousal response, we ran a series of linear mixed models (LMM) with physiological response as dependent variables (pupil dilation, skin conductance and heart rate), fixed term structure group by time by psychopathy score and random by-subject intercept.

#### Main analysis

We chose linear mixed models (LMM) to analyse the effects of affect and inter-individual differences on temporal binding, as they have several advantages over traditional ANOVAs (Matuschek et al. 2017). For example, one LMM offers a simultaneous analysis of by-participant and by-item variance, which could only be analysed in two separate ANOVAs. LMM also achieve better preservation of statistical power in models with missing data (Krueger & Tian 2004). R and the lme4 R package (Bates et al. 2014) were used to perform the analyses. The ‘lmerTest’ package in R was used to obtain the *p*-values from the LMMs via Satterthwaite’s degrees of freedom method. This method has a low Type-1 error rate, which is important when several model analyses are conducted (Kuznetsova et al. 2017). Effect sizes using Cohen’s *d* were calculated using the ‘EMAtools’ package (Kleiman 2017). Within the scope of the current analysis, Cohen’s *d* was the most appropriate given the scarce literature regarding alternative effect size measures for multilevel models and given practical challenges in estimating degrees of freedom and partitioning variance in LMMs (Brysbaert & Stevens 2018; Lorah 2018; Rights & Sterba 2018). Effect sizes for LMMs were reported as Cohen’s *d*, where 0.2 is considered a small effect size, 0.5 a medium effect and 0.8 a large effect (Cohen 1988).

The LMM analyses tested whether there was a significant difference in temporal binding (dependent variable) between the groups after watching the film clips compared to before. Including the fixed factors group and time in the model as centred contrasts was necessary, as this enables independent linear comparisons to be made between specified levels of the group and time factor (Schad et al. 2020). Therefore, in all models, one contrast compared the average binding at the time points pre vs. post (before and after watching the film clips) to account for interindividual baseline differences. A second set of contrasts was used for the fixed factor group. Repeated contrasts compared first the sexual arousal group and the neutral control group and second the calm pleasure group and the neutral control group. The psychopathic personality traits, eye blink rates and pupil dilation were entered as continuous variables and were mean-centred to allow meaningful interpretation.

Model selection of the linear mixed models was driven by theoretical considerations (Bates et al. 2015). Testing the effect of the affective induction (sexual arousal and calm pleasure) on binding required including the interaction group by time. Testing the influence of physiological arousal during the task (not during the film clips) and subjective arousal and valence response before and after each task required including the interactions group by time by arousal rating before each task, group by time by arousal rating after each task and the interactions group by time by pupil dilation and group by time by blink rates. Valence ratings before and after each task, skin conductance and heart rate were not included in the models as these measures did not differ between the groups in the manipulation check.

Testing whether inter-individual differences in psychopathy increased or reduced the effect of affect required including the interaction group by time by psychopathy in the model. Because three-way interactions were included in all models, the models failed to converge when more complex random structures than just random by-subject intercept were added to the model. Thus, random terms of all models were by subject intercept only. Model comparisons with Likelihood Ratio Tests were performed to evaluate the significance of fixed effects and are provided in the Supplementary Materials (Table 18).

First, two simple models investigating the interaction of time and group on temporal binding were performed, with one model for each binding component. In an additional model, we looked at the influence of subjective and physiological arousal by group by time and striatal dopamine levels by group by time on action binding. Then, to test inter-individual differences in psychopathy, we tested one model focusing on the interaction of time by group by psychopathy on action binding.

## Results

### Manipulation check

#### Affect induction with film clips

##### Subjective affect ratings

In total, four affective ratings were given, one before and one after each binding task. For the manipulation check, we compared the second and third rating. Mean values and SD for all four ratings are presented in Table 1. Additional analyses for all four arousal ratings can also be found in the Supplementary (Section 1.1.1).

**Table 1:** Mean values and standard deviation for affective ratings by group and time

	Pre-induction			Induction with Film Clips	Post-induction		
Group	Rating	before-task (1)	after-task (2)		before-task (3)	after-task (4)	
Neutral Control	Arousal	4.60 (1.32)	4.20 (1.75)		4.40 (1.53)	3.45 (2.04)	
	Valence	5.45 (1.43)	4.75 (1.61)		5.75 (1.51)	4.55 (1.75)	
Calm pleasure	Arousal	4.56 (1.34)	3.50 (1.57)		5.34 (1.60)	3.56 (1.74)	
	Valence	5.33 (1.56)	4.50 (1.77)		6.33 (1.76)	4.72 (1.85)	
Sexual Arousal	Arousal	4.24 (1.63)	3.48 (1.87)		6.43 (1.44)	4.29 (1.50)	
	Valence	5.52 (1.68)	4.57 (2.23)		6.33 (1.39)	4.95 (1.65)	

*Note: Range of the scale was from 1 to 9.*

###### Arousal ratings

**Fig. 2:**
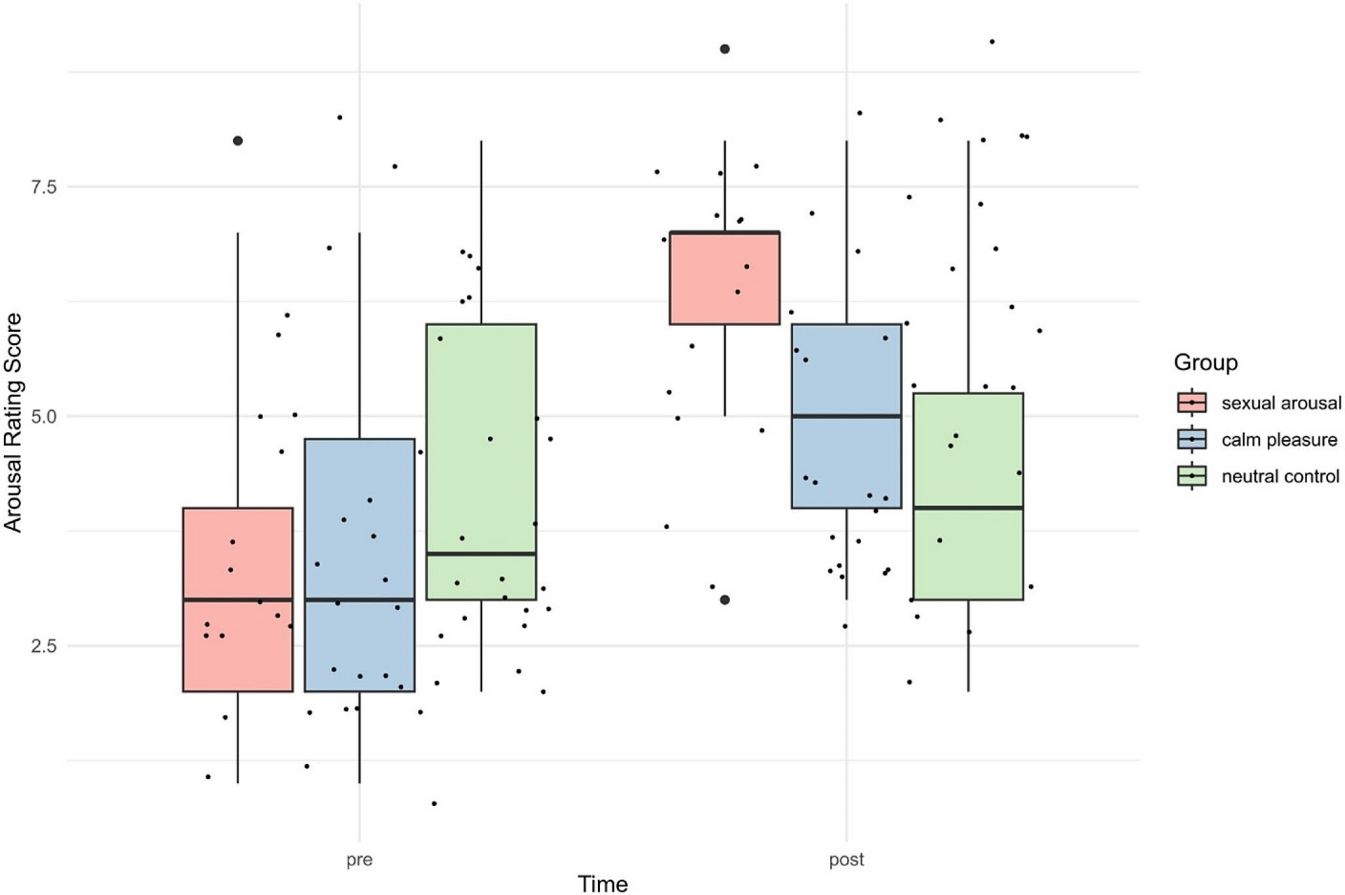
Arousal ratings on the affective grid before and after the film clips by group

In terms of the subjective arousal ratings on the affective grid, we found a large main effect of time, *F*(1,56) = 55.82, *p < .*001, η2 = .50, *95% CI* = [0.35, 1.00], no main effect group (*p = .*333) but a large effect of an interaction time by group *F*(2,56) = 13.14, *p* < .001, η2 = .32, *95% CI* =[0.15, 1.00]. Post-hoc tests confirmed higher arousal ratings after watching the film clips in the sexual arousal (*p* < .0001) and calm pleasure group (*p =* .006). Pre- and post-arousal ratings did not differ in the neutral control group (*p* = .970). As visualised in Fig. 2, post-arousal-ratings were significantly higher in the sexual arousal group compared to the neutral control (*p = .*002); there were no differences between the sexual arousal and the pleasure group, nor between the neutral control and the pleasure group (both *ps* > .2). Full post-hoc comparisons with šidák correction can be found in the Supplementary Table 3.

###### Valence ratings

In terms of the subjective valence ratings on the affective grid, we found a large main effect of main effect of time, *F*(1,56) = 45.142, *p* < .001, η2 = 0.45, *95% CI* = [0.29, 1.00]. A post-hoc test confirmed higher valence values in the post-rating compared to the pre-rating in all three groups. There was no main effect of group, nor an interaction time by group (both *p’s* > .2). The full post-hoc comparison can be found in the Supplementary Table 4.

##### Physiological arousal measures

###### Pupil dilation

**Fig. 3:**
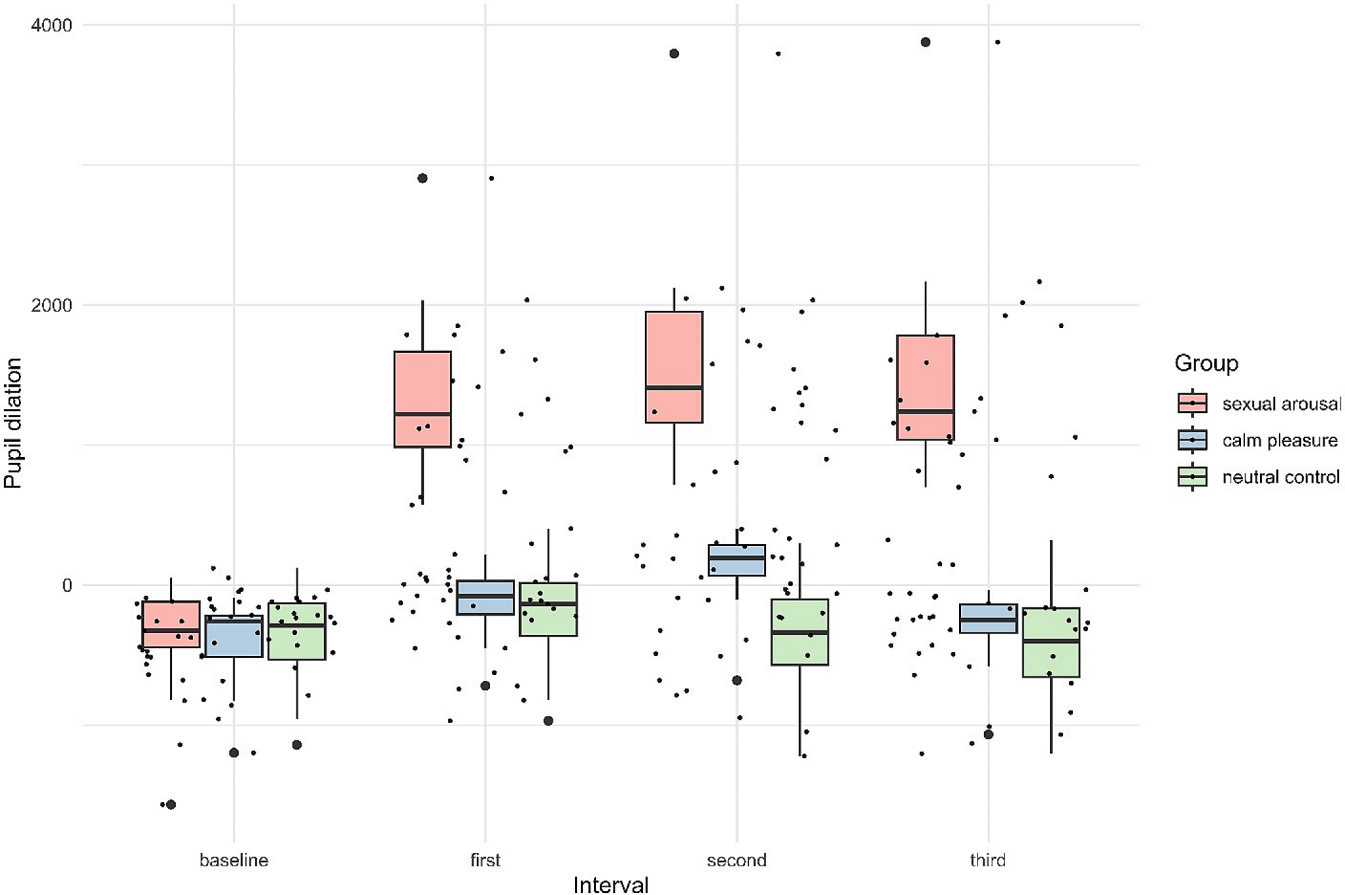
Boxplot for pupil size by group and interval. *Note*: *Sexual engagement scene started in the first interval of the sexual arousing film clip, the romantic social interaction scene started in the second interval of the calm pleasant film clip. Baseline = 500ms from film clip onset.*

For pupil, there was a large main effect of group, *F*(2,55) = 86.294, *p < .*001, η2 = 0.76, *95% CI* = [0.66, 1.00], a large main effect of interval, *F(*3, 167) = 92.39, *p < .*001, η2 = .62, *95% CI* = [0.55, 1.00], and a large interaction effect of group by interval, *F*(6,167) = 52.44, *p < .*001, η2 = .65, *95% CI* = [0.58, 1.00]. For the calm pleasant film clip, post-hoc tests confirmed significant differences between the film clip baseline and second interval (*p < .*0001), and between the second and third interval of the calm pleasant film clip (*p < .*001). The romantic social interaction took place in the second interval showing the largest pupil dilation in Fig. 3.

For the sexually arousing film clip, post-hoc tests revealed significant differences between the film clip baseline and each interval of the sexually arousing film clip (all *p’s* < .0001). As visualised in Fig. 3, in the sexual arousal group, pupils were more dilated during the film clip compared to the film clip baseline. The sexual engagement scene began during the first interval.

For the neutral control film clip, there were no differences between the film clip baseline and intervals, nor between the intervals (all *p’s* > .4). Full post-hoc comparisons with šidák correction can be found in the Supplementary Table 10.

###### Skin conductance

For skin conductance there was no main effect of group (*p* = .101), but a large main effect of interval, *F*(1, 55) = 5.086, *p = .*028, η2 = .19, *95% CI* = [0.10, 1.00]. Post-hoc tests confirmed significantly greater skin conductance in the first interval compared to the film clip baseline (*p < .*0001) and greater skin conductance during the third interval compared to the film clip baseline (*p = .*001). Skin conductance significantly dropped in the second compared to the first (*p < .*0001) and third (*p = .*003) interval of the film clip. There were no differences between film clip baseline and second interval, nor between first and third interval (both *p’s* > .7). The interaction of group by interval was also not significant (*p = .*611). Full post-hoc comparisons with šidák correction can be found in the Supplementary Table 11.

###### Heart rate

For heart rate, there was no main effect of group, no main effect of interval, nor an interaction of group by interval (all *p*’s > .7).

### Psychopathy and arousal reactivity

There were no differences in psychopathy scores between the three groups (*p = .*085). Mean values and group comparisons can be found in Supplementary, Table 15.

To examine the arousal response in psychopathy, we ran models with physiological arousal response (pupil dilation, skin conductance, heart rate) as dependent variables, fixed term structures group by time by psychopathy and random term structure participants. We present results for pupil dilation here, as it was the only variable that showed significant group differences in the manipulation check. Models for skin conductance and heart rate can be found in the Supplementary Table 16.

We found a small main effect of time, β = -97.52, *95% CI=* [-122.65, -72.39], *p* < .001, *d* = -0.141, no main effects of psychopathy (*p = .*477) but an interaction of time by psychopathy, β = -27.88, *95% CI =* [-51.89, -3.86], *p = .*023, *d* = -0.042, which had a very small effect size.

There was no main effect for sexual arousal (*p = .*834), but the interaction of time by sexual arousal was significant but very small, β = 57.59, *95% CI = [*40.63, 74.55], *p < .*001, *d =* 0.123. There was no interaction effect of sexual arousal by psychopathy (*p = .*790), and no interaction of time by sexual arousal by psychopathy (*p = .*210), suggesting that individuals high on psychopathy engaged to the same degree in the sexual arousal response as participants low on psychopathy.

There was also no main effect of calm pleasure (*p = .*473) but a very small interaction effect of time by calm pleasure, β = 167.16, *95% CI =* [111.35, 222.98], *p < .*001, *d = 0*.108). Additionally, there was no interaction effect of calm pleasure by psychopathy (*p = .*599), but an interaction of time by calm pleasure by psychopathy, β = 61.26, *95% CI =* [6.16, 116.37], *p = .*029, *d =* 0.040 which was also very small in terms of effect size, indicating that the differences in response to pleasure between individuals high and low on psychopathy were minimal.

### Effects on temporal binding

For our linear mixed models investigating the effects of sexual arousal and calm pleasure on temporal binding, we ran two models, one with dependent variable action binding, and the other one with dependent variable outcome binding. Our fixed term structure was time, group and time by group and our random term structure was participants. As already mentioned, we used the following repeated contrasts to compare the sexual arousal group versus neutral control group and neutral control group versus calm pleasure group:

Contrast 1: sexual arousal = 0.667, neutral control = 0.333, calm pleasure = 0.333

Contrast 2: sexual arousal = -0.333, neutral control = -0.333, calm pleasure = 0.667

Mean and standard deviation for action and outcome binding by group and time can be found in Table 2.

**Table 2:** Binding mean and standard deviation in milliseconds by group

		Temporal Binding	
		Pre-measure	Post-measure
Group	Binding	*M* *(SD)*	*M* *(SD)*
Neutral Control	action	14.50 (38.27)	36.15 (68.51)
	outcome	94.54 (72.26)	97.56 (105.20)
	overall	109.04 (95.63)	133.71 (115.46)
Calm pleasure	action	36.88 (78.76)	39.62 (49.40)
	outcome	151.03 (83.24)	148.79 (90.86)
	overall	213.49 (127.70)	188.40 (88.76)
Sexual Arousal	action	34.81 (51.52)	17.09 (47.66)
	outcome	94.84 (116.98)	83.44 (103.18)
	overall	129.66 (136.77)	100.52 (118.79)

#### Influence of sexual arousal and calm pleasure

**Fig. 4:**
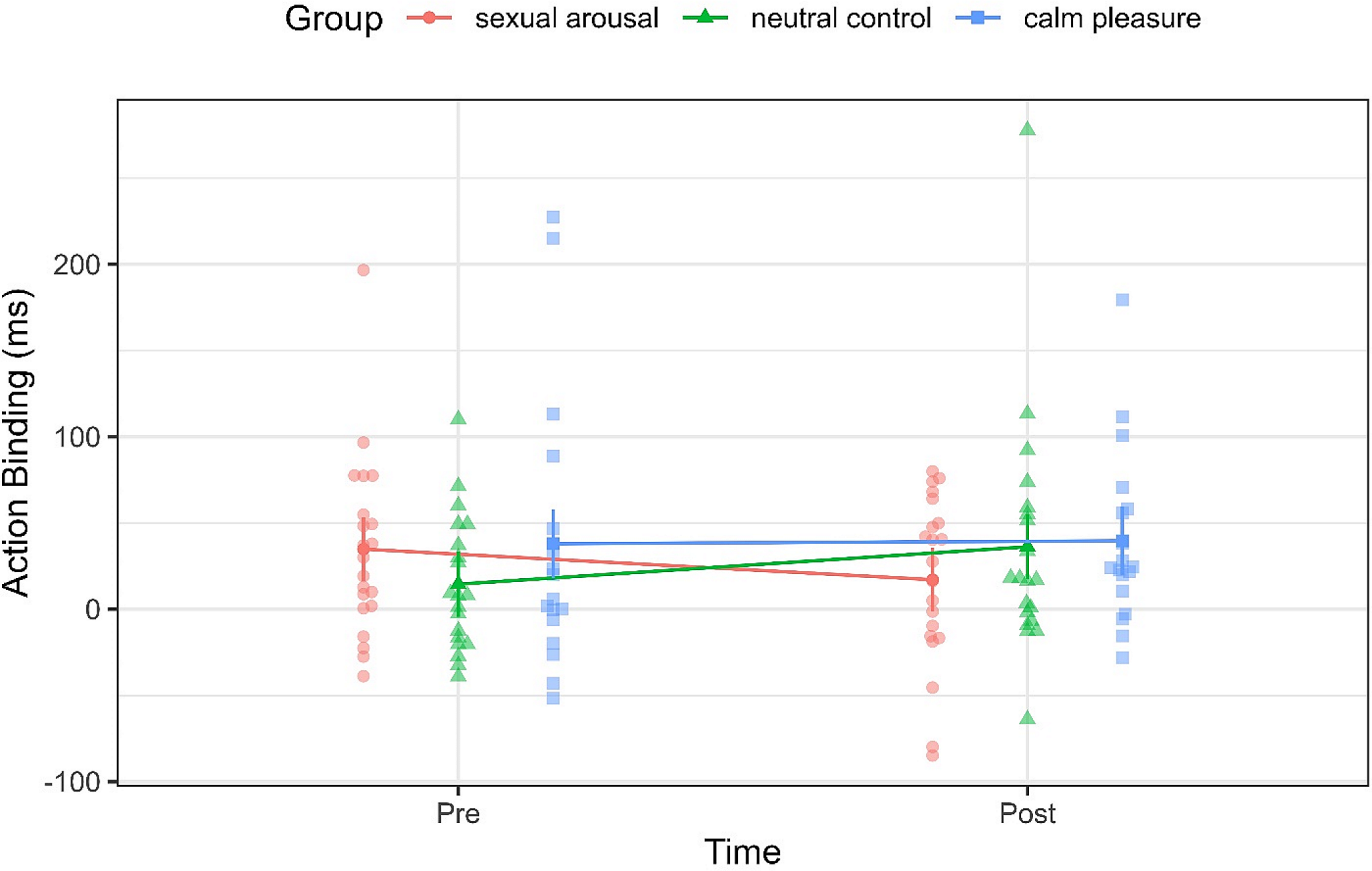
Effects of watching film clips on action binding. *Note*: *Error bars show the 95% confidence intervals.*

In the simple model for action binding, we found a small main effect of time, β = 28.53, *95% CI =* [23.94, 33.12], *p < .*001, *d =* 0.226, no significant main effect of sexual arousal (*p = .*963), but medium-sized interaction effect of time by sexual arousal, β = -39.38, *95% CI =* [ -42.57, -36.20], *p < .*001, *d =* -0.449. This indicates that action binding differed between pre- and post-measurement between the neutral control and sexually arousing group. Figure 4 visualises a decrease in action binding after watching the sexually arousing film clip and an increase in action binding after watching the neutral control clip (Fig. 4, red line compared to green line). We did also not find a main effect of pleasure (*p = .*336), but a small interaction effect of time and pleasure, β = -60.00, *95% CI =* [-70.08, -49.91], *p < .*001, *d =* -0.216. Figure 4 shows that action binding levels do not differ between pre- and post-measurement in the calm pleasure group whereas action binding increases in the neutral control group from pre- to post-measurement (Fig. 4, blue line compared to green line). Full models can be found in the Supplementary Table 13.

**Fig. 5:**
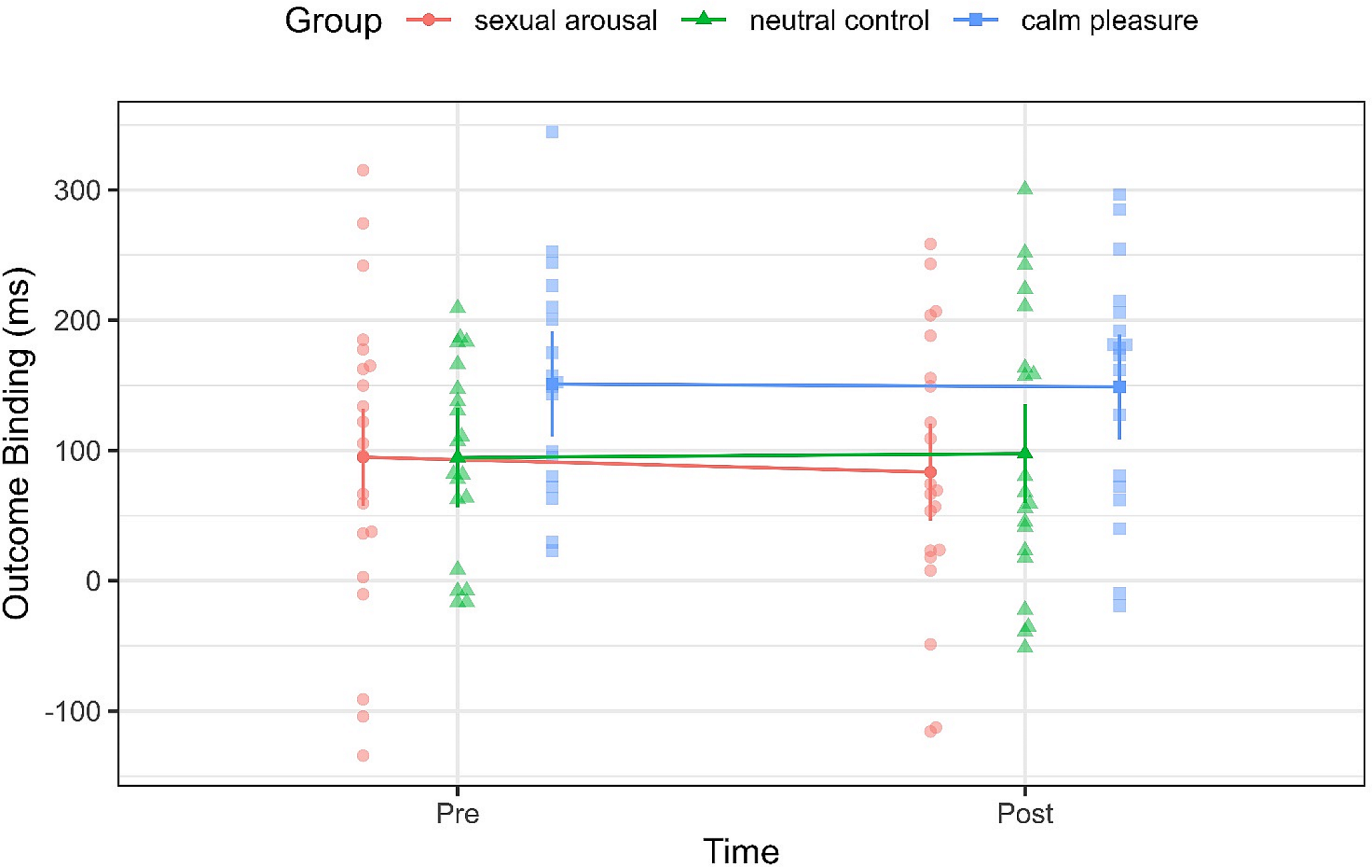
Effects of watching film clips on outcome binding. *Note*: *Error bars show the 95% confidence intervals.*

In our exploratory analyses for outcome binding we did not find a significant main effect of time (*p = .*201), no significant main effects of sexual arousal (*p = .*799), but a significant interaction of time by sexual arousal, β = -14.42, *95% CI =* [ -18.14, -10.71], *p = .*001, *d* = *-0.140*, however, this effect was very small. Figure 5 shows a decrease in outcome binding in the sexual arousal group but no change in outcome binding values in the neutral control group from pre- to post-measurement (Fig. 5, red line compared to green line).

For calm pleasure, we found a tendency of a main effect of calm pleasure, β = 161.58, *95% CI =* [ -4.67, 327.84], *p = .*057, *d* = 0.496. In Fig. 5, outcome binding seems to be higher in pre- and post-measurement in the calm pleasure group compared to the neutral control group (Fig. 5, blue line compared to green line). Additionally, we found an interaction of time by pleasure, β = -15.78, *95% CI =* [ -27.37, -4.20], *p = .*008, *d = -0.049*, but again, this effect was very small. Full models can be found in the Supplementary Table 13.

#### Interindividual differences

As our hypotheses for outcome binding were of an exploratory nature, only effects for action binding will be presented for the models examining the influences of interindividual differences. The models for outcome binding can be found in the Supplementary Tables 14 and 17.

##### Subjective and physiological arousal & striatal dopamine levels

To examine the influence of interindividual differences in physiological and subjective arousal as well as striatal dopamine levels, our simple model was extended by the fixed factor interactions group by time by arousal rating before each task (first and third rating), group by time by arousal rating after each task (second and fourth rating), group by time by pupil dilation, and group by time by blink rates.

We found no main effect of time (*p = .*634), but a main effect of arousal rating before completing each task (first and third rating), β = 5.59, *95% CI =* [3.13, 8.05], *p < .*001, *d* = 0.083 and an interaction of time by arousal rating before each task, β = -6.88, *95% CI =* [-11.72, -2.04], *p = .*005, *d* = -0.052, both of which were very small. For arousal ratings after completing each task (second and fourth rating), we found a small to medium-sized main effect, β = -24.23, *95% CI =* [-26.72, -21.74], *p < .*001, *d* = -0.353 indicating lower action binding for higher arousal ratings and an interaction effect of time by arousal after each task, β = 11.10, *95% CI =* [7.78, 14.43], *p* < .001, *d* = 0.121, that was again very small.

In terms of sexual arousal, there was a large main effect of sexual arousal, β = -88.22, *95% CI = [*-117.19, -59.24], *p < .*001, *d* = -1.385, a small interaction effect of time by sexual arousal, β = -28.95, *95% CI = [*-41.63, -16.27], *p* < .001, *d* = -0.083. Moreover, there was a small interaction effect of arousal before each task by sexual arousal, β = -4.55, *95% CI = [*-6.29, -2.81], *p < .*001, *d* = -0.095, and a small interaction effect of time by sexual arousal by arousal before each task, β = -21.59, *95% CI = [*-24.86, -18.32], *p < .*001, *d* = -0.240.

**Fig. 6:**
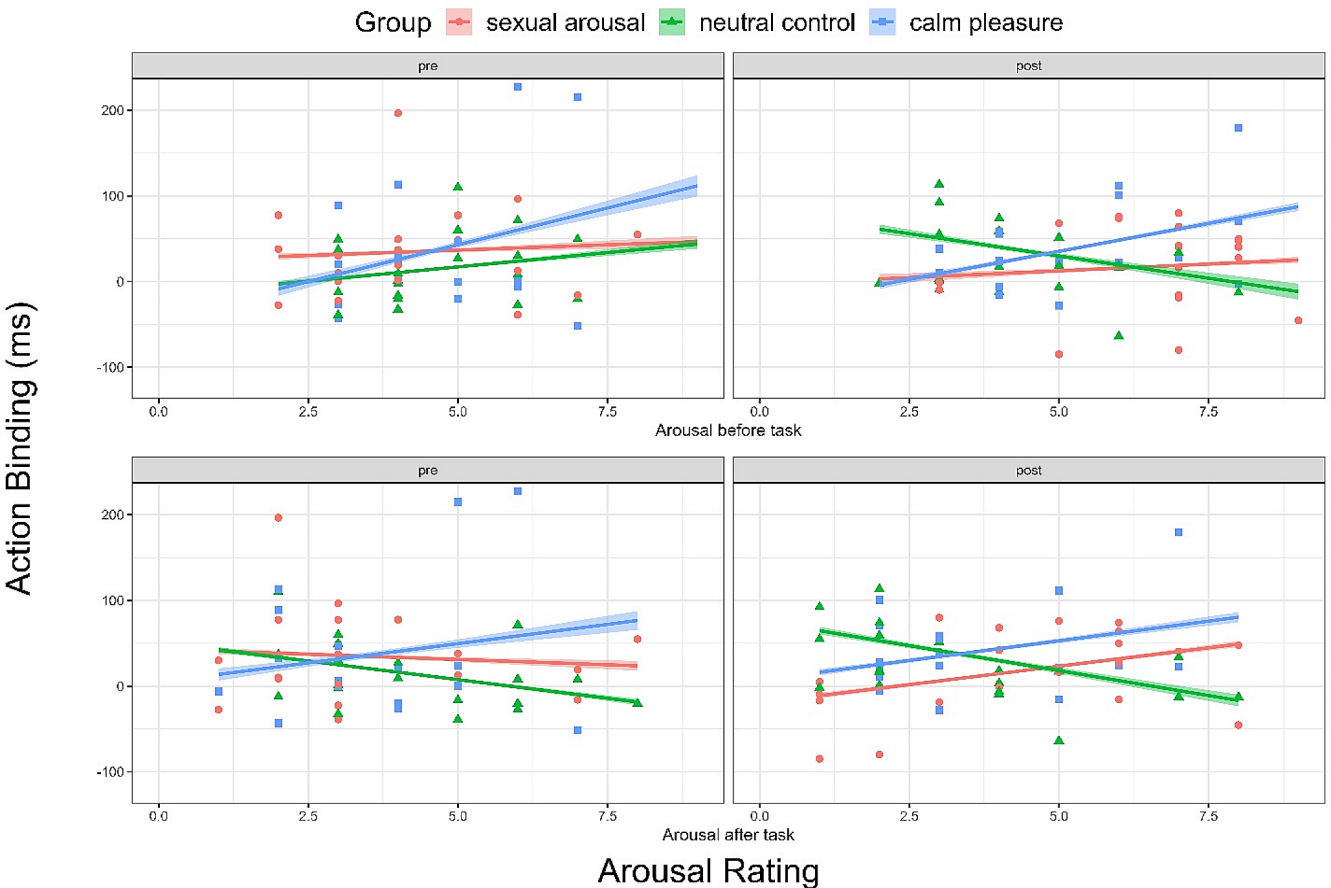
*Note*: *The top row shows the first and third arousal ratings, the bottom row shows the second and fourth arousal ratings. The shading visualises the 95% confidence intervals.*

In the top row of Fig. 6, there was no difference in action binding depending on arousal ratings before each task in the sexual arousal group. In contrast, higher arousal ratings in the neutral control group were associated with lower action binding in the post-task after watching the neutral film clip but not in the pre-task. We also found a medium-sized interaction effect of sexual arousal by arousal after each task, β = 29.51, *95% CI = [*27.69, 31.34], *p* < .001, *d* = 0.591, and a medium-sized interaction effect of time by sexual arousal by arousal rating after each task, β = 26.27, *95% CI = [*24.03, 28.51], *p < .*001, *d* = 0.426. The bottom row of Fig. 6 suggests that sexual higher arousal ratings after the post-task (the fourth rating) were associated with higher action binding, however, there was no influence of arousal ratings after the pre-task (second rating). Higher arousal ratings in the neutral control group after each task were associated with lower action binding regardless of the time of measurement.

Regarding calm pleasure, we found a large main effect of calm pleasure, β = -141.83, *95% CI = [*-231.89, -51.77], *p = .*002, *d* = -0.720. There was no interaction of time by calm pleasure (*p = .*885), no interaction of arousal before each task by calm pleasure (*p = .*570) and no interaction of time by calm pleasure by arousal after before task (*p = .*682). We did find an interaction pleasure by arousal after each task, β = 41.10, *95% CI = [*35.87, 46.33], *p < .*001, *d* = -0.108 of very small size.

As visualised in Fig. 6, higher arousal ratings after each task in the calm pleasure group seem to be consistently associated with higher action binding. There was a large interaction effect of time by calm pleasure by arousal after each task, β = -15.99, *95% CI = [*-23.56, -8.41], *p < .*001, *d* = 0.656. This can be explained by the contrasting patterns observed between the calm pleasure and neutral control groups in the bottom row of Fig. 6.

**Fig. 7:**
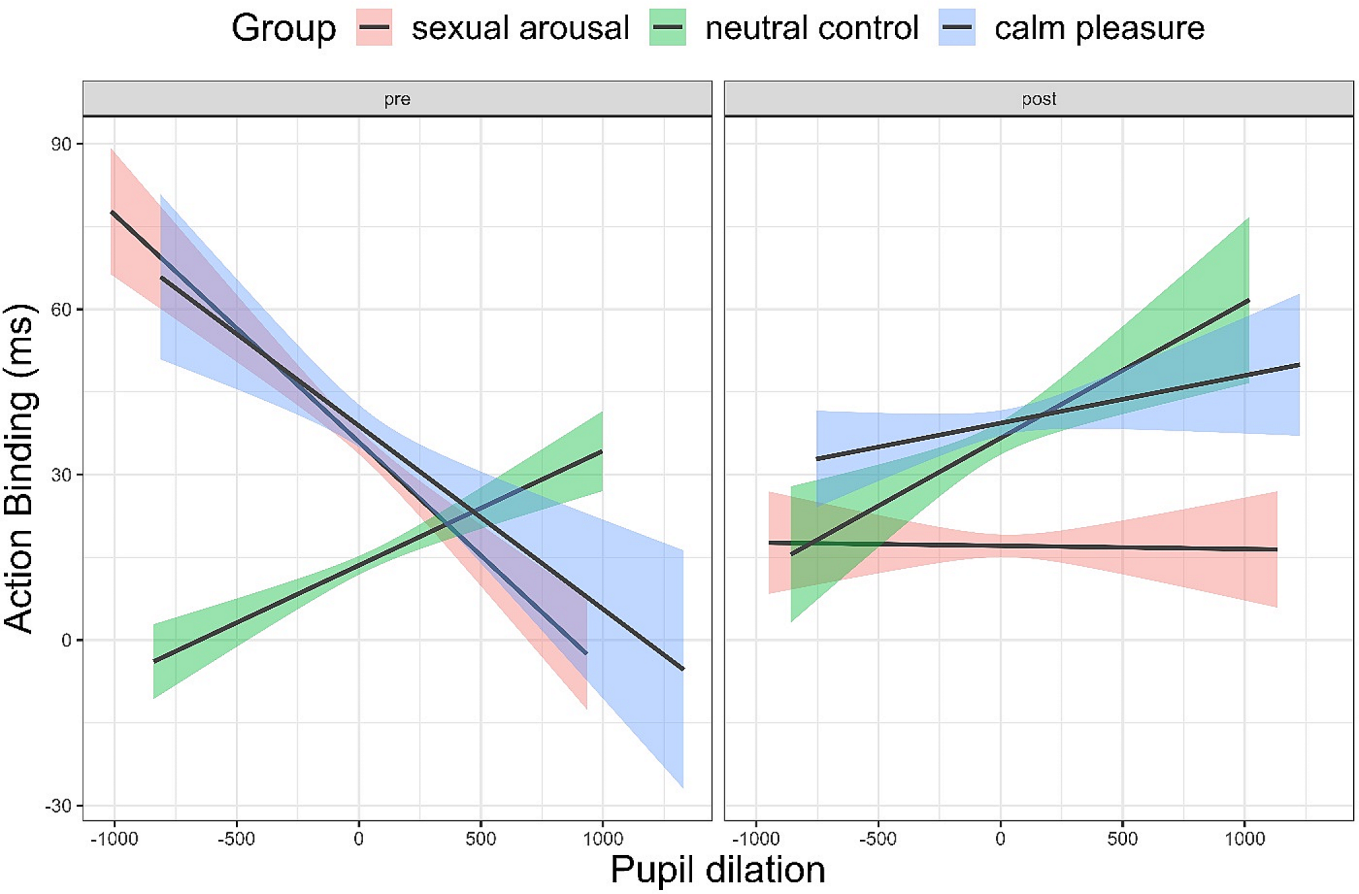
Effects of pupil dilation by group and time on action binding. *Note*: *The shading visualises the 95% confidence intervals. Pupil dilation is in arbitrary units*

With regards to our hypothesis for pupil dilation, we expected greater increases in pupil dilation to be associated with a decrease in action binding, and intermediate increases in pupil dilation to be linked to an increase in action binding. Although we did find a main effect of pupil dilation, β = -2.75, *95% CI = [*-5.12, -0.38], *p = .*023, *d* = -0.019, the effect size was close to zero. There was no interaction effect of time by pupil (*p = .*802) and no interaction of sexual arousal by pupil (*p = .*663), but a small interaction effect of time by sexual arousal by pupil β = 3.57, *95% CI = [*0.51, 6.63], *p = .*022, *d = 0*.209. Figure 7 suggests that while more dilated pupils were associated with lower action binding before the film clip, there was no difference in action binding depending on pupil dilation in the sexual arousal group after the film clip. More dilated pupils in the neutral control group were consistently associated with higher action binding. There was an interaction effect of pupil by calm pleasure, β = 7.78, *95% CI = [*2.68, 12.89], *p = .*003, *d = 0*.026, which was very small and a small to medium interaction effect of time by calm pleasure by pupil, β = 19.02, *95% CI = [*9.61, 28.44], *p < .*001, *d = 0*.306. While the neutral control group and calm pleasure group showed a similar pattern for pupil dilation on action binding during the post-task, they showed different patterns in the pre-task: the calm pleasure group had lower action binding values in more dilated pupils whereas it was reversed in the neutral control group.

**Fig. 8:**
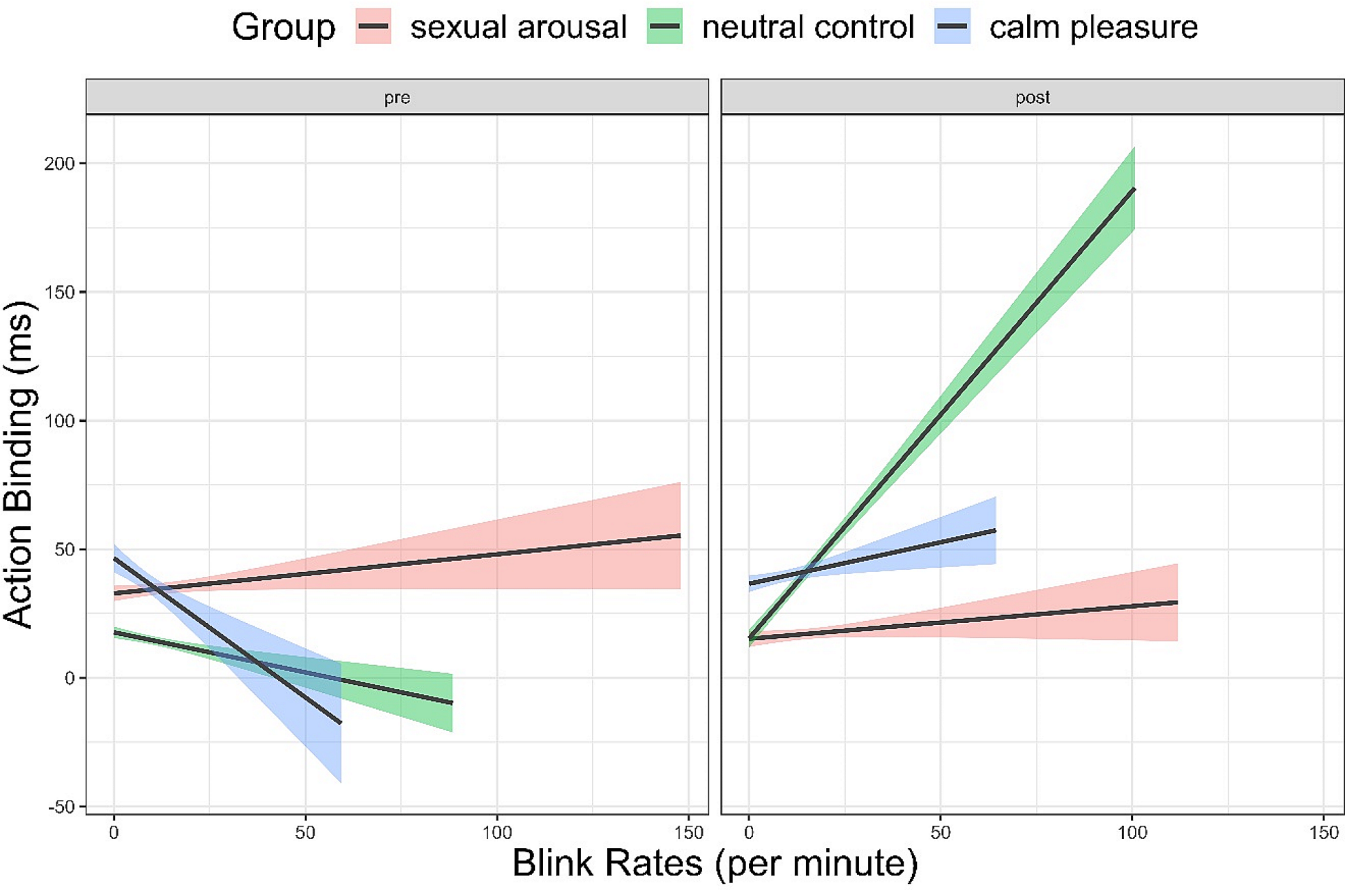
Effects of blink rates per minute by group and time on action binding. *Note*: *The shading visualises the 95% confidence intervals.*

With regards to our hypothesis for blink rates, we expected greater increases in action binding as blink rates increase, whereas greater decreases in action binding as blink rates decrease. We did find a small-sized main effect of blink rates, β = 16.83, *95% CI = [*13.62, 20.04], *p < .*001, *d = 0*.190 indicating a greater increase in action binding as blink rates increase. There was also a small to medium-sized interaction effect of time by blink rates, β = 48.25, *95% CI = [*43.45, 53.05,], *p < .*001, *d* = 0.365, an interaction effect of sexual arousal by blink rates, β = -3.42, *95% CI = [*-5.46, -1.39], *p < .*001, *d* = -0.061 that had an effect size close to zero, and small to medium-sized interaction effect of time by sexual arousal by blink rates, β = -24.29, *95% CI = [*-27.09, -21.48], *p < .*001, *d* = -0.315. While there were no differences in blink rates on action binding in the pre-test in the sexual arousal and the neutral control group, action binding increased in the post-test in the neutral control group as blink rates increased. This was not the case in the sexual arousal group. There was an interaction effect of blink rates by calm pleasure, β = -33.91, *95% CI = [*-41.14, -26.67], *p* < .001, *d* = -0.170, and an interaction effect of time by calm pleasure by blink rates, β = -84.15, *95% CI = [*-95.85, -72.46], *p < .*001, *d* =-0.262, both of which were small sized. Figure 8 shows a smaller range of blink rates in the calm pleasure group compared to the neutral group and the sexual arousal group. The full model can be found in the Supplementary Table 14.

##### Psychopathy

To examine the influence of interindividual differences in psychopathic traits, our simple model for action binding was extended by the fixed factor interaction group by time by psychopathy score.

We found no main effect of psychopathy (*p = .*680) but a main effect of time, β = 17.90, *95% CI = [*13.15,22.64], *p < .*001, *d = 0*.137, and an interaction of time by psychopathy, β = 6.38, *95% CI = [*1.87,10.89], *p = .*006, d = 0.051, however, both of which were very small.

**Fig. 9:**
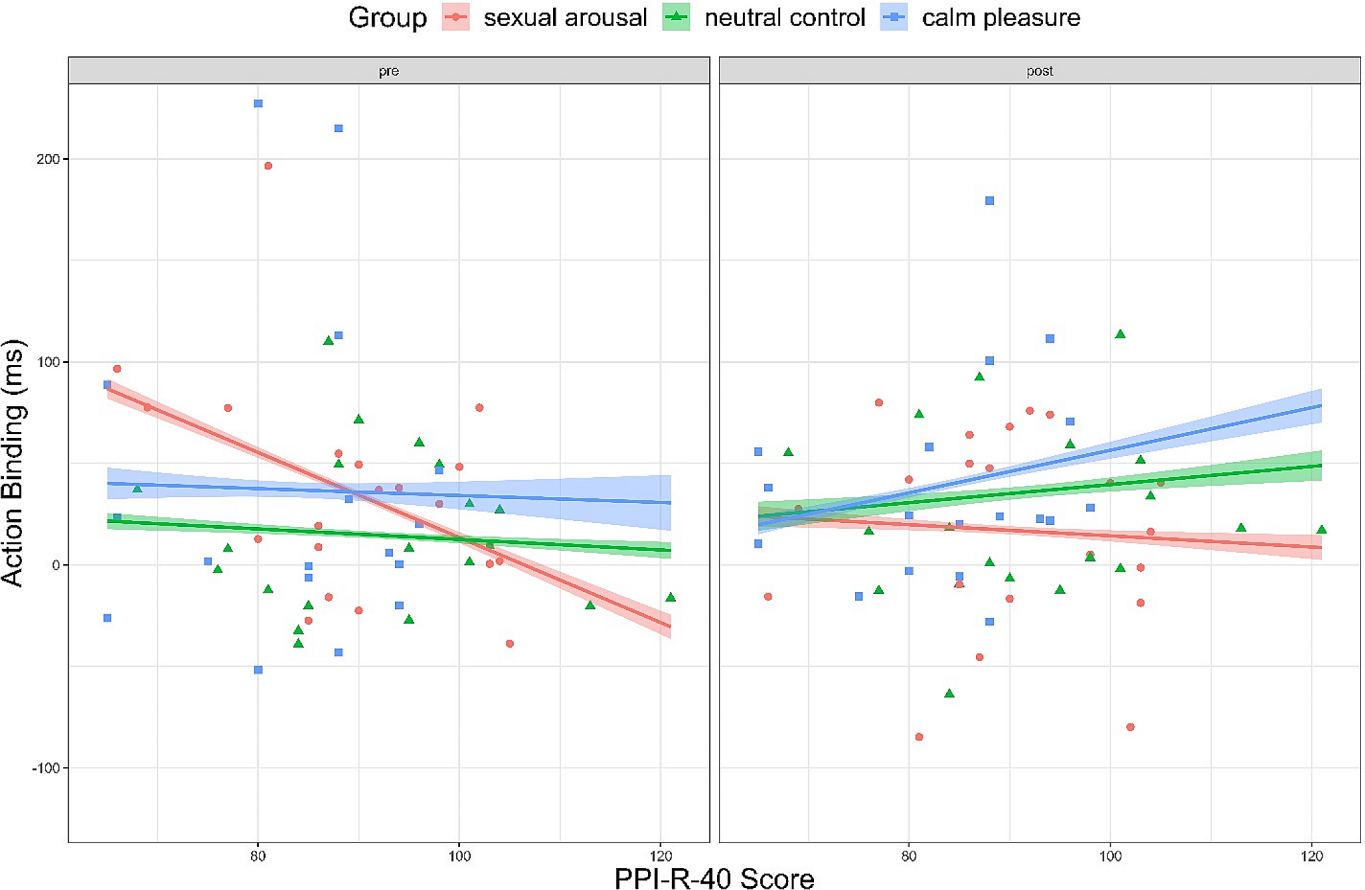
Action binding before (pre) and after (post) the film clips by group as a factor of psychopathy. *Note*: *The shading visualises the 95% confidence intervals*

We found no main effect of sexual arousal (*p = .*879), no interaction of sexual arousal and psychopathy (*p = .*257) but a medium-sized interaction effect of sexual arousal and time, β = -38.63, *95% CI = [*-41.81, -35.44], *p* < .001, *d* = -0.441, as well as a small interaction effect of time by sexual arousal by psychopathy, β = 13.20, *95% CI = [*10.05,16.35], *p < .*001, *d = 0*.153. Figure 9 shows that action binding increased in individuals high on psychopathy regardless of what film clip they watched. For individuals low on psychopathy, action binding was reduced after watching the sexually arousing film clip (at post-measurement). In contrast, action binding in the control group did not differ between pre- and post-measurement for individuals low on psychopathy.

For pleasure, we found no main effect of pleasure (*p = .*281) and only a very small-sized interaction of calm pleasure and time, β = -34.68, *95% CI=* [-45.28, -24.09], *p < .*001, *d* = -0.119. Further, we found no interaction of calm pleasure by psychopathy (*p = .*787) and only a very small-sized interaction effect of time by calm pleasure by psychopathy, β = 19.08, *95% CI=* [-8.72, 29.43], *p < .*001, *d = 0*.067. In Fig. 9, action binding is increased in the calm pleasure group and the neutral control group at post-measurement in individuals high on psychopathy (Fig. 9, blue line compared to green line). For individuals low on psychopathy action binding did not differ between pre- and post-measurement in the calm pleasure group, nor in the neutral control group. The full model can be found in the Supplementary Table 17.

## Discussion

In the current study, we investigated the influence of positive affect differing in arousal intensity—sexual arousal and calm pleasure—and the additional influence of inter-individual differences on temporal binding. While we had specific hypotheses for action binding, effects on outcome binding were tested exploratory.

We found evidence for reduced action binding in sexual arousal but no effects for calm pleasure. When taking the inter-individual arousal response into account, subjectively perceived arousal had a larger influence when given after the task compared to when given before the task. Moreover, high arousal after the task was associated with reduced action binding. The relationship between pupil dilation and action binding was complex and differed between the groups. Higher blink rates were associated with enhanced action binding.

Although individuals high on psychopathy showed the same affective response on a physiological level, they still seemed to be less affected in their action binding in sexual arousal and profited more from a pleasant state.

### State: influence of sexual arousal and calm pleasure

The evidence for our first hypothesis was mixed. Although we did find a medium-sized effect of reduced action binding in response to the sexually arousing film clip, the interpretation of this effect is restricted by an increase in action binding in response to the neutral control film clip. The manipulation check showed an increase in subjective valence ratings and greater skin conductance during the neutral control film compared to the baseline but no changes in subjective arousal ratings, nor pupil dilation or heart rate. In terms of pleasure, we found no evidence for the postulated facilitation effect on action binding. Exploratory analysis for outcome binding indicated small effects of reduced outcome binding in response to the sexually arousing film clip compared to the neutral control film clip. There was no influence of the calm pleasant induction on outcome binding.

These results extend previous work in several important ways. First, we reported effects of affect for both action binding and outcome binding, while many previous studies focused on valence effects on outcome binding (Gentsch et al. 2015; Takahata et al. 2012; Yoshie and Haggard 2013,2017) or reported results for temporal binding as a sum score (Wen et al. 2015). Several recent studies have indicated that action and outcome binding could be uncorrelated (Siebertz & Jansen 2022; Tonn et al. 2021). Thus, binding in paradigms relying on the sum of the two could confound the underlying mechanisms (Siebertz & Jansen 2022) and may not be helpful in understanding the processes. In fact, findings from a meta-analysis (Tanaka et al. 2019) found that action binding depends on whether one can control outcome onsets with voluntary actions–highlighting the importance of retrospective, inferential and bottom-up processes. In contrast, outcome binding shifts depend more on the degree to which participants can predict the outcome onset–highlighting the importance of predictive, prospective, and top-down processes (Tanaka et al. 2019). Therefore, our results are in agreement with the hypothesis that action binding and outcome binding are uncorrelated (Siebertz & Jansen 2022; Tonn et al. 2021), rely on different mechanisms (Tanaka et al. 2019), and therefore vary differentially with emotional arousal.

Following Christensen et al. (2019), we studied how an emotional state influenced temporal binding, rather than the perception of events that were themselves emotionally significant. We provide the first evidence that positive states influence action binding as a function of arousal. While positive states with low arousal and medium arousal may be beneficial, states with high arousal seem to reduce binding extending previous findings of small or absent effects of positive compared to neutral states on temporal binding (Gentsch & Synofzik 2014; Takahata et al. 2012; Yoshie & Haggard 2013).

### State and trait: affect and inter-individual differences

Furthermore, alterations of the sense of agency in positive affective states seem to vary as a function of inter-individual differences. We modelled the effects of inter-individual differences in subjectively perceived and physiologically experienced arousal and in striatal dopamine levels on action binding in one model for our second hypothesis and effects for psychopathy in another model for our third hypothesis.

#### Striatal dopamine levels

In line with our hypothesis for striatal dopamine levels, we found a small main effect of blink rates showing a greater increase in action binding as blink rates increased. In previous research, positive priming was reported to have a facilitating effect on outcome binding in individuals with higher dopaminergic activity measured via blink rates (Aarts et al. 2012), we were able to extend these for action binding. It seems that the dopaminergic system’s involvement in temporal binding only plays a role in neuropsychiatric disorders (for a review see, Moccia et al. 2024) but also for healthy subjects (Aarts et al. 2012; Moore et al. 2011).

Graham et al. (2015) have pointed out that when interpreting the effects of age and psychosis-like experiences on temporal binding, dopamine may actually modulate the integration window in which two events can be bound (Albrecht et al. 2011; Seitz & Dinse 2007), particularly in motor-perceptual learning (Hosp et al. 2011; Molina-Luna et al. 2009). Graham et al also drew a connection to research that postulates that dopamine activity functions as an internal pacemaker. Research using manipulations with dopaminergic medication (e.g. selective dopamine blockers) was able to show that dopamine alters the speed of the internal clock, predominantly for intervals less than 500ms (Buhusi & Meck 2002; Meck 1996; Rammsayer 2009). Dopamine has also been extensively studied in the context of reward prediction error and reinforcement learning (Diederen & Fletcher 2021; Lerner et al. 2021) to explain how the brain processes rewards and updates its expectations based on outcomes. When an unexpected reward is received, dopamine neurons fire more vigorously, leading to an increase in dopamine release. Conversely, when an expected reward is omitted or the outcome is worse than expected, dopamine neuron activity decreases, resulting in a reduction in dopamine release. Our design was highly predictable as the tone was played 250ms after the key press in the agency action trials. This encouraged prediction and hence dopamine release facilitated learning and anticipation effects for binding.

#### Physiological and subjective arousal

We further assumed that great increases in pupil dilation—such as in sexual arousal—would be associated with a decrease in action binding, whereas intermediate increases in pupil dilation—such as in calm pleasure—would be linked to an increase in action binding. Although we did find a main effect of pupil size indicating that more dilated pupils were associated with reduced action binding, this effect size was close to zero. When looking at the interactions of time by group by pupil size, it seems that there is some evidence that more dilated pupils were associated with higher action binding after watching the calm pleasant, and the neutral control film clip. However, action binding was independent of pupil size after watching the sexually arousing film clip, which is not in line with our hypothesis. Potentially, physical arousal may facilitate action binding up to a certain point from which it either reverses or loses its effects. It also needs to be considered that pupil dilation not only tracks arousal but also cognitive load or exertion (e.g., van der Wel & van Steenbergen 2018). Although the temporal binding task is not difficult, the length and monotony put a strain on participants that confounds pupil size effects.

Regarding subjective arousal ratings, we observed a small main effect suggesting that higher arousal ratings were associated with enhanced action binding when the arousal rating was given before the task (first and third arousal rating). However, the effect was larger for arousal ratings that were given after the task (second and fourth rating): here, higher arousal ratings were associated with reduced action binding. In contrast to the physiological measures that were tracked during the task, the affective ratings were given with a retrospective or prospective bias and did not actually reflect the participant’s affective state during the task, which may explain the differences between arousal ratings given before and after the task and between subjective ratings and physiological measures.

#### Psychopathy

For our third hypothesis, we investigated the role of psychopathy as a personality trait postulated to show alterations in the affective response. For participants high on psychopathy, we hypothesized increased action binding, however, we did not find a main effect of psychopathy on action binding. Recent findings (Schwarz et al. 2022) suggest that individual factors only increased the explained variance for differences in the sense of agency by a few points of percentage, which could explain our unexpected results. Schwarz et al (2022) identified other personality constructs correlated with explicit sense of agency ratings: gender identity, neuroticism, and openness, all of which are not core traits of psychopathy.

In exploratory analyses, we examined whether psychopathy may lessen the effects of sexual arousal but intensify the effects pleasure since higher psychopathy is correlated with a lower autonomous nervous system response and a hypersensitive reward system. Our analyses revealed that although psychopathy did not moderate differences in response to arousal and valence, psychopathic traits played a role in the reduction of the sexual arousal effects and the facilitation effects of pleasure, resulting in increased action binding in individuals higher on psychopathy.

These interaction effects of situation and personality have also been explored in the aforementioned study by Schwarz et al., who found that individual factors explained up to 10% of the variance in situations of highest uncertainty. The participants’ change in sense of agency ratings were most affected by assertiveness, self-esteem, and neuroticism. With regards to our effects, it would have been interesting to see whether our effects would have been stronger in a less predictable/more uncertain condition in the temporal binding task.

In terms of the affective response, individuals high on psychopathy have engaged in the affective response to the same degree as participants low on psychopathy. According to the motivational framework by Groat and Shane (2020), this may be due to the intrinsic incentive—positive states are rewarding in themselves—that positive states offer compared to negative states. At the same time, individuals high on psychopathy seemed to be less affected by sexual arousal and profited more from pleasure than individuals low on psychopathy. This dissociation between physiological arousal response, behaviour, and cognition could be due to differences in emotion regulation strategies (Walker et al. 2022) or differences in emotion goals (Spantidaki Kyriazi et al. 2021).

### Limitations and future research

#### Increase in action binding in control group

A complication in our findings is that we observed an increase in valence ratings (from rating two after the pre-task to rating 3 after the film clip) in the neutral group along with an increase in action binding after watching the control film clip, which is difficult to explain. The same effect had been found in a previous experiment with a different control film clip (Render & Jansen 2020). This could either reflect an anticipation or learning effect caused by the design as we used the same predictable interval of 250ms between key press and tone in the agency trials. Another possibility is that it was elicited by the neutral control film clip, however, in a pilot study, the neutral control film clip received a neutral valence rating (please see Supplementary: Pre-test Film Clips). Another explanation is that the neutral control film clip was received particularly better in valence in contrast to performing the temporal binding task. The absolute difference between the first baseline valence rating and the third valence rating (after the film clip) is smaller than between the second and the third valence rating. Nonetheless, the difference is significant and shows higher valence ratings after the film clip compared to the initial baseline rating rejecting this explanation (please see Tables 5 and 6 Supplementary). Either way, the increase in the neutral control group’s action binding is problematic, as it restricts the interpretation of results. To clarify this effect, groups could receive different control treatments, such as taking a break without watching a film clip or performing an easy working memory task.

#### Affect induction

The affect induction with the three film clips was partially successful. While there was an increase in arousal and valence in the sexual arousal group in subjective ratings and an increase in pupil size and skin conductance, there was also the already mentioned unexpected increase in valence ratings and skin conductance in the neutral control group. Yet, the distinct feature between these two groups is the arousal level, as confirmed by differences in the subject arousal ratings and pupil dilation between the groups.

For calm pleasure, we did find an increase not only in valence ratings but also in arousal ratings which was unexpected. An increase in arousal was also confirmed on a physiological level in skin conductance and pupil dilation. However, arousal ratings and pupil dilation indicated lower arousal than in the sexual arousal group and higher arousal than in the neutral control group. Unfortunately, the missing group difference in valence ratings restricted the interpretation of the effects between the calm pleasure group and the neutral control group in terms of the effects of valence on binding. Hence, although the three groups did not differ in valence, they did differ in arousal level: low, medium and high. As a consequence, we have not included valence in our analysis and only interpreted the effects of calm pleasure as a medium-level arousal state.

As the temporal binding tasks were fairly long (about 25 minutes per task) it seems unlikely that the affect induction with the film clips would have lasting effects over the course of the tasks. Mean and SD in Table 1 and our additional analyses in the Supplementary Section 1.1.1. confirm a significant drop in arousal ratings from the third to fourth rating in the sexual arousal and calm pleasure group. In addition to that, the effects of arousal ratings given after performing the temporal binding task seemed to have a stronger influence on action binding. Considering that the affective manipulation may not have lasted for the full duration of the task, this emphasizes the strength of the sexual arousal effects on action binding, as we consistently found in all models. Effects of calm pleasure may have played a more significant role in a different design such as when including explicit sense of agency measures.

#### Multi method approach for sense of agency

Recent work has directly assessed the influence of affective manipulations on implicit and explicit measures of SOA (Kaiser et al. 2021). It was found that affective manipulations have a much more consistent impact on explicit than implicit measures: Positive compared to negative action outcomes increase the explicit feeling of agency. Our study did not include any explicit measures of the sense of agency, which may partly explain the mixed evidence for our hypothesis. Moreover, although using implicit measures gives several advantages over explicit self-report measures (for review, see Jensen et al. 2015) it also comes with limitations. As mentioned in the introduction, temporal binding suffers from methodological problems such as poor correlations between temporal binding and explicit agency measures or the lack of correlations to other implicit measures (e.g., Dewey & Knoblich 2014). Since these methods may not measure the same thing, the relevance of implicit measures for social concepts of responsibility is less clear (Christensen et al. 2019). One could argue that the reliabilities for the binding measures in the Libet clock paradigm are higher than for other binding measures such as interval estimation and that the Libet clock offers to investigate action and outcome binding separately (Siebertz & Jansen 2022; Tanaka et al. 2019).

#### Future research

Another limiting factor is the small sample size. Since some effects in our study are relatively small, replication with an additional larger sample, especially in populations with higher scores of psychopathy, would be valuable.

As our study was merely focused on two affective states and two individual characteristics, the generalisability of our results is limited. Experiments with more systematic manipulations could clarify the effects. An interesting modification could be to integrate additional affective states. Fear and anger—both high arousal states—have already been examined by Christensen et al. (2019); low arousal states such as sadness remain to complete the cluster for the two valence and arousal dimensions. Previous research suggests that sadness biases recognition memory toward negatively valenced words, and impairs facial emotion recognition, but not general cognitive processes (Chepenik et al. 2007). Others claim broader benefits of negative affect for cognition, motivation, and interpersonal behaviour (Forgas 2014).

### Significance of the findings

Keeping these limitations in mind, our findings indicate that participants experienced the linkage between action and outcome less strongly under certain affective states. But this result should not be overstated—a reduced temporal binding does not imply that participants lost their sense of agency or acted against their own will (Christensen et al. 2019).

Investigating the architecture of volitional processes is important because concepts of legal responsibility are based on a common understanding of the fully functional sense of agency (Haggard 2017; Haggard & Tsakiris 2009; Moore 2016). In most Western countries, exemptions from legal responsibility are based on incapacity and mental illness clauses (Malatesti et al. 2020). The term incapacity connotes that the agent was incapable of knowing that the act was wrong while committing the crime. Thus, the distinction between culpable and nonculpable offenders is based on their decision-making abilities during the act. Given that psychopathy is known to be responsible for a disproportionate amount of crime and violence, and is associated with high rates of future offending (Douglas et al. 2019), studying the sense of agency in arousing states in this personality trait is of utmost importance. Our study adds to this discourse of legal responsibility, providing evidence that action binding and outcome binding are two separate mechanisms that are each determined by one’s individual response to arousal and pleasure. This can build the basis for research that investigates how to separately evaluate those two mechanisms from a legal perspective.

## Conclusion

Our results provide preliminary evidence that sexual arousal reduces temporal binding in a similar way as threat or frustration do. Exploratory analyses also revealed that inter-individual differences in subjectively perceived and physiologically experienced arousal and striatal dopamine levels might predict the effects of positive affective states. Although participants high on psychopathy engaged to the same degree in the emotional response, they seemed to be more resilient to the effects of that emotional response on action binding. This indicates the potential impacts of state and trait arousal characteristics on situational expectations about the sense of agency. Given the implications for the legal responsibility discourse at a societal level, the findings suggest the need for further investigation into how one’s sense of agency is altered by affective state and personality.

### Electronic Supplementary Material

Below is the link to the electronic supplementary material

Supplementary Material 1

## Data Availability

Data set and code is available on OSF (https://osf.io/pskmh)

## Declarations

### Deviations from pre-registration

Originally, we planned to use the 3-minute baselines at the beginning of the experiment to correct spontaneous eye blink rates. However, as inter-individual differences are controlled for by the pre-post-design of the study, we have dropped this additional baseline correction.
