# Supplementary material for: Arousal, interindividual differences and temporal binding a psychophysiological study
Source: Psychol Res. 2024 May 28;88(5):1653–77. doi: 10.1007/s00426-024-01976-3 (PMC11282159; doi:10.1007/s00426-024-01976-3)
Supplement: Supplementary file 1 — Supplementary Material 1 [file 426_2024_1976_MOESM1_ESM.docx]

# Supplementary Materials

## Pre-test Film Clips

Eleven participants rated all three film clips with the affective grid on a scale from 1 to 9 for valence and arousal. The sexually arousing film clip was a threesome scene from the film Love (2015) by Gaspar Noe and was rated *M* = 6.11 (*SD* = 2.09) in valence and *M* = 7.89 (*SD* = .93) in arousal. The calm pleasant film clip showed the opening and closing scenes of the film Pride and Prejudice (2005) by Joe Wright, a film clip used in other studies to induce calm but happy states ((Gabert-Quillen et al., 2015) on a scale 1 to 8: calmness (*M* = 6.3, SD = 1.9), happiness (*M* = 4.4 *SD* = 2.4), (Ramzan et al., 2016) on scale of 1 to 5: valence close to 4, arousal close to 1.5)). In our pre-test survey it was rated *M* = 7.00 (*SD* = 2.18) in valence and *M* = 4.78 (*SD* = 2.28) in arousal. The emotionally neutral film clip was naturalistic footage of a pedestrian street scene, and was rated *M* = 4.36 (*SD* = 1.57) in valence and *M* = 3.64 (*SD* = 3.64) in arousal. A one-way ANOVA with post hoc tests using Bonferroni correction confirmed a main effect of valence for the calm pleasant film clip compared to the neutral film clip, *F*(2, 26) = 4.851, *p* = .005, but only showed a tendency for the sexually arousing film clip (*p* = .055). A main effect of arousal was found as well, *F*(2, 26) = 13.62, showing higher arousal for the sexually arousing film clip (*p* <.001) compared to the calm pleasant and neutral film clip. The calm pleasant and neutral film clip did not differ from one another on rated arousal.

## Manipulation check

## Self-Report – Affective Grid

**Table 3**

*P-values for post-hoc comparison with šidák correction for Arousal ratings with within factor time.*

| Group contrast | Time | estimate | SE | df | t.ratio | p.value |
| --- | --- | --- | --- | --- | --- | --- |
| Neutral control – calm pleasure | post | -0.9333 | 0.558 | 89.9 | -1.671 | .4619 |
| Neutral control – sexual arousal | post | -20.286 | 0.537 | 89.9 | -3.777 | .0017 |
| Calm pleasure – sexual arousal | post | -10.952 | 0.552 | 89.9 | -1.984 | .2664 |
| Neutral control – calm pleasure | pre | 0.7000 | 0.558 | 89.9 | 1.254 | .7629 |
| Neutral control – sexual arousal | pre | 0.7238 | 0.537 | 89.9 | 1.348 | .6984 |
| Calm pleasure – sexual arousal | pre | 0.0238 | 0.552 | 89.9 | 0.043 | 1.000 |
| Time contrast | Group | estimate | SE | df | t.ratio | p.value |
| Post - pre | Neutral control | 0.20 | 0.386 | 56 | 0.518 | .9389 |
| Post - pre | Calm pleasure | 1.83 | 0.407 | 56 | 4.509 | .0001 |
| Post - pre | Sexual arousal | 2.95 | 0.376 | 56 | 7.843 | <.0001 |

**Table 4**

*P-values for post-hoc comparison with šidák correction for Valence ratings with within factor time.*

| contrast | estimate | SE | df | t.ratio | p.value |
| --- | --- | --- | --- | --- | --- |
| Post - pre | 1.53 | 0.228 | 56 | 6.733 | <.0001 |

- - 1. **Additional analysis for all four affect ratings**

**Table 5**

*Repeated measures ANOVA for Arousal Ratings with between factor group and time of rating number*

| Factor | F | *df* | *p* |
| --- | --- | --- | --- |
| group | 0.623 | 2,56 | .54 |
| Time of Rating | 21.864 | 3,168 | <.001 |
| Group*Time of rating | 5.014 | 6,168 | <.001 |

**Table 6**

*P-values for post-hoc comparison with šidák correction for Arousal ratings with by group by time of rating.*

| Rating number contrast | group | estimate | SE | df | t.ratio | p.value | |
| --- | --- | --- | --- | --- | --- | --- | --- |
| four – one | Calm pleasure | -10.000 | 0.429 | 168 | -2.332 | 0.3159 |  |
| four - three | Calm pleasure | -17.778 | 0.429 | 168 | -4.146 | 0.0010 |  |
| four - two | Calm pleasure | 0.0556 | 0.429 | 168 | 0.130 | 1 |  |
| one - three | Calm pleasure | -0.7778 | 0.429 | 168 | -1.814 | 0.7367 |  |
| one - two | Calm pleasure | 10.556 | 0.429 | 168 | 2.462 | 0.2358 |  |
| three - two | Calm pleasure | 18.333 | 0.429 | 168 | 4.276 | 0.0006 |  |
| four - one | Neutral control | -11.500 | 0.407 | 168 | -2.827 | 0.0907 |  |
| four - three | Neutral control | -0.9500 | 0.407 | 168 | -2.336 | 0.3137 |  |
| four - two | Neutral control | -0.7500 | 0.407 | 168 | -1.844 | 0.7128 |  |
| one - three | Neutral control | 0.2000 | 0.407 | 168 | 0.492 | 1 |  |
| one - two | Neutral control | 0.4000 | 0.407 | 168 | 0.983 | 0.9992 |  |
| three - two | Neutral control | 0.2000 | 0.407 | 168 | 0.492 | 1 |  |
| four - one | Sexual arousal | 0.0476 | 0.397 | 168 | 0.120 | 1 |  |
| four - three | Sexual arousal | -21.429 | 0.397 | 168 | -5.398 | <.0001 |  |
| four - two | Sexual arousal | 0.8095 | 0.397 | 168 | 2.039 | 0.5465 |  |
| one - three | Sexual arousal | -21.905 | 0.397 | 168 | -5.518 | <.0001 |  |
| one - two | Sexual arousal | 0.7619 | 0.397 | 168 | 1.919 | 0.6498 |  |
| second - two | Sexual arousal | 29.524 | 0.397 | 168 | 7.438 | <.0001 | |

Note*: P value adjustment: sidak method for 18 tests*

**Table 7**

*Repeated measures ANOVA for Valence Ratings with between factor group and rating number*

| Factor | F | *df* | *p* |
| --- | --- | --- | --- |
| group | 0.148 | 2,56 | .862 |
| time | 21.485 | 3,168 | <.001 |
| Group*time | 0.634 | 6,168 | .703 |

**Table 8**

*P-values for post-hoc comparison with šidák correction for Valence ratings with within factor time of rating.*

| Rating number contrast | estimate | SE | df | t.ratio | p.value | |
| --- | --- | --- | --- | --- | --- | --- |
| four - one | -0.694 | 0.215 | 168 | -3.229 | 0.0089 |  |
| four - three | -1.397 | 0.215 | 168 | -6.500 | <.0001 |  |
| four - three | 0.134 | 0.215 | 168 | 0.625 | 0.9896 |  |
| one - three | -0.703 | 0.215 | 168 | -3.271 | 0.0078 |  |
| one - two | 0.829 | 0.215 | 168 | 3.854 | 0.0010 |  |
| one - two | 1.532 | 0.215 | 168 | 7.125 | <.0001 |  |

Note: *Results are averaged over the levels of: group, P value adjustment: šidák method for 6 tests*

- 1. **Physiological Measures**
     1. **During Film clips**

**Table 9**

*Mean (SD) for physiology measures by Group and film clip interval.*

|  |  | | | Group | |  | |  |
| --- | --- | --- | --- | --- | --- | --- | --- | --- |
| Physiology measure | | Interval | Neutral Control | | Calm pleasure | Sexual arousal | Total |  |
| Pupillometry | | Baseline |  | |  |  |  |  |
|  | | Beginning | -216.82 (357.13) | | -122.61 (222.56) | 1322.22 (548.63) | 359.72 (826.04) |  |
|  | | Middle | -388.39 (403.47) | | 141.12 (252.72) | 1552.94 (671.82) | 464.14 (969.49) |  |
|  | | End | -425.38 (427.00) | | -291.25 (242.75) | 1446.83 (703.27) | 281.92 (1006.65) |  |
|  | | Total | -343.53 (400.76) | | -90.91 (295.86) | 1440.67 (641.30) |  |  |
| SCR | | Baseline |  | |  |  |  |  |
|  | | Beginning | 1.78 (1.45) | | 1.65 (1.66) | 2.69 (2.80) | 2.08 (2.12) |  |
|  | | Middle | 0.07 (2.46) | | -0.69 (2.02) | 1.36 (2.40) | 0.31 (2.42) |  |
|  | | End | 0.99 (1.59) | | 1.66 (3.08) | 1.34 (1.73) | 1.33 (2.19) |  |
|  | | Total | 0.95 (1.98) | | 0.87 (2.55) | 1.80 (2.40) |  |  |
| HR | | Beginning | -3.35 (7.14) | | -4.14 (7.22) | -4.19 (6.33) | -3.89 (6.78) |  |
|  | | Middle | 1.57 (3.43) | | 2.39 (2.20) | 1.94 (3.01) | 1.95 (2.92) |  |
|  | | End | 1.32 (2.38) | | -0.76 (1.95) | 1.11 (1.58) | 0.61 (2.16) |  |
|  | | Total | -0.152 (5.22) | | -0.836 (5.17) | -0.381 (4.91) |  |  |

*Note: Film clips are divided in three equally long intervals beginning, middle and end. Baseline = 500ms from film clip onset. Pupil dilation is in arbitrary units*

**Table 10**

*P-values for post-hoc comparison with šidák correction for pupil dilation by group and interval.*

| Film interval contrast | group | estimate | SE | df | t.ratio | p.value |
| --- | --- | --- | --- | --- | --- | --- |
| baseline - beginning | Neutral control | -164 | 98.7 | 167 | -1.658 | .8474 |
| baseline - middle | Neutral control | 13 | 97.0 | 167 | 0.134 | 1.000 |
| baseline - end | Neutral control | 50 | 97.0 | 167 | 0.515 | 1.000 |
| beginning - middle | Neutral control | 177 | 98.7 | 167 | 1.790 | .7554 |
| beginning - end | Neutral control | 214 | 98.7 | 167 | 2.165 | .4414 |
| middle - end | Neutral control | 37 | 97.0 | 167 | 0.381 | 1.000 |
| baseline - beginning | Calm pleasure | -291 | 102.3 | 167 | -2.847 | .0858 |
| baseline - middle | Calm pleasure | -553 | 102.3 | 167 | -5.403 | <.0001 |
| baseline - end | Calm pleasure | -120 | 102.3 | 167 | -1.176 | .9930 |
| beginning - middle | Calm pleasure | -261 | 102.3 | 167 | -2.556 | .1875 |
| beginning - end | Calm pleasure | 171 | 102.3 | 167 | 1.671 | .8396 |
| middle - end | Calm pleasure | 432 | 102.3 | 167 | 4.227 | .0007 |
| baseline - beginning | Sexual arousal | -1701 | 94.7 | 167 | -17.958 | <.0001 |
| baseline - middle | Sexual arousal | -1918 | 94.7 | 167 | -20.256 | <.0001 |
| baseline - end | Sexual arousal | -1812 | 94.7 | 167 | -19.135 | <.0001 |
| beginning - middle | Sexual arousal | -218 | 94.7 | 167 | -2.298 | .3401 |
| beginning - end | Sexual arousal | -111 | 94.7 | 167 | -1.177 | .9930 |
| middle - end | Sexual arousal | 106 | 94.7 | 167 | 1.120 | .9960 |

Note: *P value adjustment: šidák method for 18 tests. Film clips are divided in three equally long intervals beginning, middle and end. Baseline = 500ms from film clip onset.*

**Table 11**

*P-values for post-hoc comparison with šidák correction for skin conductance with within factor interval.*

| Film clip interval contrast | estimate | SE | df | t.ratio | p.value |
| --- | --- | --- | --- | --- | --- |
| baseline - beginning | -19.781 | 0.386 | 167 | -5.129 | <.0001 |
| baseline - middle | -0.0966 | 0.383 | 167 | -0.252 | .9999 |
| baseline - end | -14.641 | 0.383 | 167 | -3.818 | .0011 |
| beginning - middle | 18.815 | 0.386 | 167 | 4.879 | <.0001 |
| beginning - end | 0.5140 | 0.386 | 167 | 1.333 | .7057 |
| middle - end | -13.675 | 0.383 | 167 | -3.566 | .0028 |

Note: *P value adjustment: šidák method for 6 tests.* *Film clips are divided in three equally long intervals beginning, middle and end Baseline = 500ms from film clip onset.*

- - 1. **During the Task**

**Table 12**

*Mean (SD) for physiology measures by Group and Interval for baseline, pre and post task.*

|  |  | | | Group | |  | |  |
| --- | --- | --- | --- | --- | --- | --- | --- | --- |
| Physiology measure | | Interval | Neutral Control | | Calm pleasure | Sexual arousal | Total |  |
| Pupillometry | | Baseline | -291 (273) | | -150. (259) | -327. (425) | -261 (335) |  |
|  | | Pre | 46.2 (225) | | 47.5 (221) | 26.4 (202) | -39.5 (216) |  |
|  | | post | -18.1 (211) | | 38.4 (214) | 23.0 (219) | 13.8 (216) |  |
| SCR | |  |  | |  |  |  |  |
|  | | Baseline | 1.93 (1.84) | | 1.94 (2.08) | 2.94 (4.43) | 2.31 (3.08) |  |
|  | | Pre | 0.858 (1.72) | | 0.888 (1.89) | 0.706 (1.31) | 0.811 (1.64) |  |
|  | | post | 0.724 (1.57) | | 0.867 (1.45) | 1.21 (3.03) | 0.951 (2.22) |  |
| HR | |  |  | |  |  |  |  |
|  | | Baseline | -3.48 (6.50) | | -0.92 (3.80) | -5.02 (6.78) | -3.21 (6.04) |  |
|  | | Pre | -1.71 (5.05) | | -1.52 (5.16) | -1.10 (4.67) | -1.44 (4.96) |  |
|  | | post | -1.21 (5.66) | | -1.51 (5.42) | -1.03 (5.06) | -1.24 (5.38) |  |
| Blink Rates | |  |  | |  |  |  |  |
|  | | Baseline | 13.2 (11.8) | | 13.9 (10.6) | 16.4 (14.8) | 14.6 (12.5) |  |
|  | | Pre | 10.3 (11.6) | | 8.48 (8.15) | 13.3 (14.3) | 10.8 (11.9) |  |
|  | | post | 12.0 (15.4) | | 9.64 (9.66) | 15.2 (13.1) | 12.4 (13.2) |  |

Note: *Baseline= first three minutes of experiment. Pupil dilation is in arbitrary units*

**Figure 10**

*Skin conductance and Pupil dilation by group by trial.*


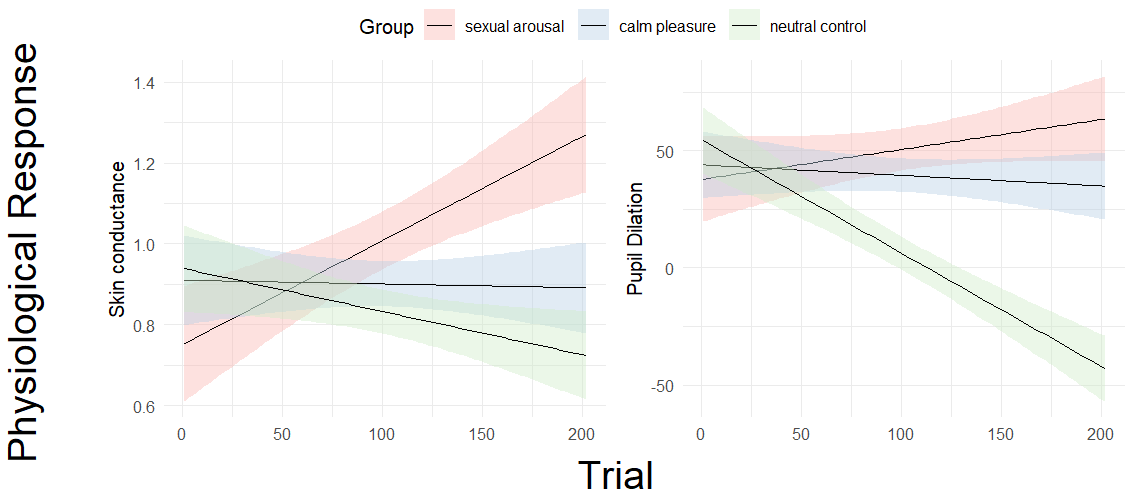


Note: *Trial 1 -101 are part of the pre-binding task, trial 103-202 are part of the post-binding task. Trial 102 (film clips) is not included. Pupil dilation is in arbitrary units.*

- 1. **Main Analysis: Tables for Linear mixed models**

**Table 13**

*Linear mixed models with repeated contrasts.*

*Dependent variable = action binding or outcome binding,*

*fixed term structure = time, group, time by group*

*random term structure = participants*

1. Repeated contrasts: sexual arousal vs. neutral control and neutral control vs. calm pleasure

*Contrast 1:* sexual arousal = 0.6666667, neutral control = 0.3333333, calm pleasure = 0.3333333

*Contrast 2:* sexual arousal = -0.3333333, neutral control = -0.3333333, calm pleasure = 0.6666667

|  | **Action Binding** | | | **Outcome Binding** | | |
| --- | --- | --- | --- | --- | --- | --- |
| *Predictors* | *Estimates* | *CI* | *p* | *Estimates* | *CI* | *p* |
| (Intercept) | 12.07 | -25.65 – 49.79 | .530 | 39.89 | -36.35 – 116.12 | .305 |
| Time (pre vs. post) | 28.53 | 23.94 – 33.12 | **<.001** | 3.47 | -1.84 – 8.78 | .201 |
| Group (neutral control vs. calm pleasure) | 40.39 | -41.88 – 122.65 | .336 | 161.58 | -4.67 – 327.84 | .057 |
| Group (sexual arousal vs. neutral control) | 0.62 | -25.74 – 26.99 | .963 | -6.91 | -60.20 – 46.38 | .799 |
| Time (pre vs. post) × Group (neutral control vs. calm pleasure) | -60.00 | -70.08 – -49.91 | **<.001** | -15.78 | -27.37 – -4.20 | **.008** |
| Time (pre vs. post) × Group (sexual arousal vs. neutral control) | -39.38 | -42.57 – -36.20 | **<.001** | -14.42 | -18.14 – -10.71 | **<.001** |
| **Random Effects** | | | | | | |
| σ^2^ | 1352.32 | | | 1839.03 | | |
| τ_00_ | 1846.94 _Participant_ | | | 7563.31 _Participant_ | | |
| ICC | 0.58 | | | 0.80 | | |
| N | 59 _Participant_ | | | 59 _Participant_ | | |
| Observations | 11700 | | | 11800 | | |
| Marginal R^2^ / Conditional R^2^ | 0.032 / 0.591 | | | 0.071 / 0.818 | | |

**Table 14**

*Linear mixed models with repeated contrasts.*

*Dependent variable = action binding or outcome binding,*

*fixed term structure = time, group, time by group, pupil dilation, pupil dilation by time, pupil dilation by group, pupil dilation by time by group, blink rates, blink rates by time, blink rates by group, blink rates by time by group, arousal ratings before each task, arousal ratings before each task by time, arousal ratings before each task by group, arousal ratings before each task by time by group, arousal ratings after each task, arousal ratings after each task by time, arousal ratings after each task by group, arousal ratings after each task by time by group*

*random term structure = participants*

1. Repeated contrasts: sexual arousal vs. neutral control and neutral control vs. calm pleasure

*Contrast 1:* sexual arousal = 0.6666667, neutral control = 0.3333333, calm pleasure = 0.3333333

*Contrast 2:* sexual arousal = -0.3333333, neutral control = -0.3333333, calm pleasure = 0.6666667

|  | **Action Binding** | | | **Outcome Binding** | | |
| --- | --- | --- | --- | --- | --- | --- |
| *Predictors* | *Estimates* | *CI* | *p* | *Estimates* | *CI* | *p* |
| (Intercept) | 85.79 | 44.82 – 126.75 | **<.001** | -95.35 | -177.43 – -13.26 | **.023** |
| Time (pre vs. post) | 4.54 | -14.12 – 23.20 | .634 | -221.34 | -241.18 – -201.50 | **<.001** |
| Group (neutral control vs. calm pleasure) | -141.83 | -231.89 – -51.77 | **.002** | 474.80 | 295.40 – 654.20 | **<.001** |
| Group (sexual arousal vs. neutral control) | -88.22 | -117.19 – -59.24 | **<.001** | 105.08 | 47.48 – 162.68 | **<.001** |
| pupil dilation | -2.75 | -5.12 – -0.38 | **.023** | -1.34 | -3.89 – 1.21 | .302 |
| Blink rates | 16.83 | 13.62 – 20.04 | **<.001** | -4.18 | -7.63 – -0.73 | **0.018** |
| Arousal rating before each task | 5.59 | 3.13 – 8.05 | **<.001** | 16.36 | 13.72 – 19.01 | **<.001** |
| Arousal rating after each task | -24.23 | -26.72 – -21.74 | **<.001** | 13.39 | 10.70 – 16.07 | **<.001** |
| Time (pre vs. post) × Group (neutral control vs. calm pleasure) | 2.96 | -36.99 – 42.92 | .885 | 515.10 | 473.20 – 557.01 | **<.001** |
| Time (pre vs. post) × Group (sexual arousal vs. neutral control) | -28.95 | -41.63 – -16.27 | **<.001** | 83.48 | 69.83 – 97.14 | **<.001** |
| Time (pre vs. post) × pupil dilation | -0.55 | -4.83 – 3.74 | .802 | -37.09 | -41.69 – -32.50 | **<.001** |
| Group (neutral control vs. calm pleasure) × pupil dilation | 7.78 | 2.68 – 12.89 | **0.003** | 3.89 | -1.60 – 9.38 | .164 |
| Group (sexual arousal vs. neutral control) × pupil dilation | 0.37 | -1.31 – 2.06 | .663 | 0.02 | -1.79 – 1.84 | .981 |
| Time (pre vs. post) × Blink rates | 48.25 | 43.45 – 53.05 | **<.001** | -1.73 | -6.82 – 3.36 | .505 |
| Group (neutral control vs. calm pleasure) × Blink rates | -33.91 | -41.14 – -26.67 | **<.001** | 15.83 | 8.07 – 23.60 | **<.001** |
| Group (sexual arousal vs. neutral control) × Blink rates | -3.42 | -5.46 – -1.39 | **.001** | -2.62 | -4.81 – -0.43 | **.019** |
| Time (pre vs. post) × Arousal rating before each task | -6.88 | -11.72 – -2.04 | **.005** | 89.03 | 83.83 – 94.23 | **<.001** |
| Group (neutral control vs. calm pleasure) × Arousal rating before each task | 1.63 | -3.99 – 7.24 | .570 | -45.69 | -51.73 – -39.66 | **<.001** |
| Group (sexual arousal vs. neutral control) × Arousal rating before each task | -4.55 | -6.29 – -2.81 | **<.001** | -23.71 | -25.58 – -21.83 | **<.001** |
| Time (pre vs. post) × Arousal rating after each task | 11.10 | 7.78 – 14.43 | **<.001** | -49.00 | -52.58 – -45.43 | **<.001** |
| Group (neutral control vs. calm pleasure) × Arousal rating after each task | 41.10 | 35.87 – 46.33 | **<.001** | -16.86 | -22.48 – -11.23 | **<.001** |
| Group (sexual arousal vs. neutral control) × Arousal rating after each task | 29.51 | 27.69 – 31.34 | **<.001** | 4.11 | 2.14 – 6.08 | **<.001** |
| (Time (pre vs. post) × Group (neutral control vs. calm pleasure)) × pupil dilation | 19.02 | 9.61 – 28.44 | **<.001** | 84.99 | 74.93 – 95.04 | **<.001** |
| (Time (pre vs. post) × Group (sexual arousal vs. neutral control)) × pupil dilation | 3.57 | 0.51 – 6.63 | **.022** | 19.03 | 15.74 – 22.33 | **<.001** |
| (Time (pre vs. post) × Group (neutral control vs. calm pleasure)) × Blink rates | -84.15 | -95.85 – -72.46 | **<.001** | 33.50 | 21.19 – 45.80 | **<.001** |
| (Time (pre vs. post) × Group (sexual arousal vs. neutral control)) × Blink rates | -24.29 | -27.09 – -21.48 | **<.001** | -13.08 | -16.09 – -10.06 | **<.001** |
| (Time (pre vs. post) × Group (neutral control vs. calm pleasure)) × Arousal rating before each task | 2.09 | -7.91 – 12.10 | .682 | -223.30 | -234.01 – -212.59 | **<.001** |
| (Time (pre vs. post) × Group (sexual arousal vs. neutral control)) × Arousal rating before each task | -21.59 | -24.86 – -18.32 | **<.001** | -58.41 | -61.94 – -54.89 | **<.001** |
| (Time (pre vs. post) × Group (neutral control vs. calm pleasure)) × | -15.99 | -23.56 – -8.41 | **<.001** | 147.16 | 139.03 – 155.28 | **<.001** |
| (Time (pre vs. post) × Group (sexual arousal vs. neutral control)) × Arousal rating after each task | 26.27 | 24.03 – 28.51 | **<.001** | 52.52 | 50.11 – 54.93 | **<.001** |
| **Random Effects** | | | | | | |
| σ^2^ | 1016.20 | | | 1177.06 | | |
| τ_00_ | 1962.83 _Participant_ | | | 8522.33 _Participant_ | | |
| ICC | 0.66 | | | 0.88 | | |
| N | 59 _Participant_ | | | 59 _Participant_ | | |
| Observations | 11675 | | | 11775 | | |
| Marginal R^2^ / Conditional R^2^ | 0.257 / 0.746 | | | 0.172 / 0.900 | | |

## Personality characteristics

Four one-way ANOVAs with between subject factor reward induction and within subject factor participant for age and psychopathy confirmed, that the three groups only differed in age but not in psychopathy or baseline blink rates (table 8).

**Table 15**

*Mean, SD and Group Comparison for Age, Psychopathy Score and baseline Blink Rates by Group.*

| Variable | Group | | | Group comparison | |
| --- | --- | --- | --- | --- | --- |
|  | Neutral Control | Calm Pleasure | Sexual Arousal | *F*(2,56) | *p* |
| Age | 22.35(3.57) | 24.06(3.23) | 24.81(5.15) | 3.771 | .026 |
| Psychopathy | 92.35(12.76) | 83.94(10.31) | 89.81(11.09) | 2.584 | .085 |
| Blink rates (baseline) | 13.22(11.76) | 13.89(10.61) | 16.43(14.85) | 0.366 | .695 |

**Table 16**

Arousal reactivity in Psychopathy

*Linear mixed models with repeated contrasts.*

*Dependent variable = pupil dilation, skin conductance, or heart rate*

*fixed term structure = time, group, psychopathy, time by group, time by psychopathy, group by psychopathy, time by group by psychopathy,*

*random term structure = participants*

1. Repeated contrasts: sexual arousal vs. neutral control and neutral control vs. calm pleasure

*Contrast 1:* sexual arousal = 0.6666667, neutral control = 0.3333333, calm pleasure = 0.3333333

*Contrast 2:* sexual arousal = -0.3333333, neutral control = -0.3333333, calm pleasure = 0.6666667

|  | **Pupil dilation** | | | **Skin Conductance** | | | **Heart Rate** | | |
| --- | --- | --- | --- | --- | --- | --- | --- | --- | --- |
| *Predictors* | *Estimates* | *CI* | *p* | *Estimates* | *CI* | *p* | *Estimates* | *CI* | *p* |
| (Intercept) | -3.55 | -94.44 – 87.34 | .939 | 0.76 | -0.02 – 1.55 | .056 | -1.02 | -2.23 – 0.20 | .101 |
| Time (pre vs. post) | -97.52 | -122.65 – -72.39 | **<.001** | -0.25 | -0.49 – -0.00 | **.047** | 0.99 | 0.32 – 1.66 | **.004** |
| Group (neutral control vs. calm pleasure) | 73.94 | -127.93 – 275.82 | .473 | 0.25 | -1.49 – 1.98 | .779 | -0.90 | -3.58 – 1.78 | .510 |
| Group (sexual arousal vs. neutral control) | 6.57 | -54.77 – 67.91 | .834 | 0.16 | -0.37 – 0.69 | .548 | 0.40 | -0.42 – 1.23 | .338 |
| Psychopathy Score (PPI) | -31.49 | -118.31 – 55.33 | .477 | 0.41 | -0.35 – 1.16 | .292 | 0.44 | -0.71 – 1.58 | .453 |
| Time (pre vs. post) × Group (neutral control vs. calm pleasure) | 167.16 | 111.35 – 222.98 | **<.001** | 0.95 | 0.41 – 1.49 | **.001** | -1.96 | -3.43 – -0.48 | **.009** |
| Time (pre vs. post) × Group (sexual arousal vs. neutral control) | 57.59 | 40.63 – 74.55 | **<.001** | 0.65 | 0.49 – 0.82 | **<.001** | -0.44 | -0.89 – 0.01 | .057 |
| Time (pre vs. post) × Psychopathy Score (PPI) | -27.88 | -51.89 – -3.86 | **.023** | 0.06 | -0.18 – 0.30 | .642 | 0.55 | -0.08 – 1.18 | .085 |
| Group (neutral control vs. calm pleasure) × Psychopathy Score (PPI) | 53.44 | -145.85 – 252.74 | .599 | -1.15 | -2.87 – 0.57 | .191 | -1.52 | -4.15 – 1.10 | .255 |
| Group (sexual arousal vs. neutral control) × Psychopathy Score (PPI) | 8.23 | -52.44 – 68.90 | .790 | 0.09 | -0.43 – 0.61 | 0.736 | -0.10 | -0.92 – 0.71 | .808 |
| (Time (pre vs. post) × Group (neutral control vs. calm pleasure)) × Psychopathy Score (PPI) | 61.26 | 6.16 – 116.37 | **.029** | 0.01 | -0.56 – 0.57 | 0.981 | -1.34 | -2.78 – 0.10 | .068 |
| (Time (pre vs. post) × Group (sexual arousal vs. neutral control)) × Psychopathy Score (PPI) | 10.73 | -6.05 – 27.51 | .210 | -0.00 | -0.16 – 0.16 | 0.989 | 0.15 | -0.30 – 0.60 | .510 |
| **Random Effects** | | | | | | | | | |
| σ^2^ | 36699.10 | | | 3.25 | | | 25.10 | | |
| τ_00_ | 9440.63 _Participant_ | | | 0.68 _Participant_ | | | 1.54 _Participant_ | | |
| ICC | 0.20 | | | 0.17 | | | 0.06 | | |
| N | 59 _Participant_ | | | 58 _Participant_ | | | 57 _Participant_ | | |
| Observations | 11775 | | | 11273 | | | 11369 | | |
| Marginal R^2^ / Conditional R^2^ | 0.014 / 0.215 | | | 0.017 / 0.188 | | | 0.006 / 0.063 | | |

**Table 17**

*Linear mixed models for psychopathy with two different sets of orthogonal contrasts.*

*Dependent variable = action binding or outcome binding,*

*fixed term structure = time, group, psychopathy, time by group, time by psychopathy, group by psychopathy, time by group by psychopathy,*

*random term structure = participants*

1. Repeated contrasts: sexual arousal vs. neutral control and neutral control vs. calm pleasure

*Contrast 1:* sexual arousal = 0.6666667, neutral control = 0.3333333, calm pleasure = 0.3333333

*Contrast 2:* sexual arousal = -0.3333333, neutral control = -0.3333333, calm pleasure = 0.6666667

|  | **Action Binding** | | | **Outcome Binding** | | |
| --- | --- | --- | --- | --- | --- | --- |
| *Predictors* | *Estimates* | *CI* | *p* | *Estimates* | *CI* | *p* |
| (Intercept) | 9.71 | -29.47 – 48.89 | .627 | 55.68 | -23.03 – 134.40 | .166 |
| Time (pre vs. post) | 17.90 | 13.15 – 22.64 | **<.001** | 16.34 | 10.89 – 21.79 | **<.001** |
| Group (neutral control vs. calm pleasure) | 47.91 | -39.11 – 134.93 | .281 | 115.94 | -58.89 – 290.78 | .194 |
| Group (sexual arousal vs. neutral control) | 2.05 | -24.39 – 28.49 | .879 | -9.95 | -63.07 – 43.18 | .714 |
| Psychopathy Score (PPI) | -7.88 | -45.31 – 29.54 | .680 | 29.68 | -45.51 – 104.87 | .439 |
| Time (pre vs. post) × Group (neutral control vs. calm pleasure) | -34.68 | -45.28 – -24.09 | **<.001** | -57.90 | -70.01 – -45.80 | **<.001** |
| Time (pre vs. post) × Group (sexual arousal vs. neutral control) | -38.63 | -41.81 – -35.44 | **<.001** | -10.94 | -14.61 – -7.26 | **<.001** |
| Time (pre vs. post) × Psychopathy Score (PPI) | 6.38 | 1.87 – 10.89 | **.006** | 47.59 | 42.38 – 52.79 | **<.001** |
| Group (neutral control vs. calm pleasure) × Psychopathy Score (PPI) | 11.85 | -74.05 – 97.76 | .787 | -81.56 | -254.16 – 91.03 | .354 |
| Group (sexual arousal vs. neutral control) × Psychopathy Score (PPI) | -15.13 | -41.28 – 11.02 | .257 | 23.80 | -28.74 – 76.34 | .375 |
| (Time (pre vs. post) × Group (neutral control vs. calm pleasure)) × Psychopathy Score (PPI) | 19.08 | 8.72 – 29.43 | **<.001** | -141.55 | -153.50 – -129.61 | **<.001** |
| (Time (pre vs. post) × Group (sexual arousal vs. neutral control)) × Psychopathy Score (PPI) | 13.20 | 10.05 – 16.35 | **<.001** | -22.66 | -26.29 – -19.02 | **<.001** |
| **Random Effects** | | | | | | |
| σ^2^ | 1297.29 | | | 1729.25 | | |
| τ_00_ | 1781.75 _Participant_ | | | 7209.75 _Participant_ | | |
| ICC | 0.58 | | | 0.81 | | |
| N | 59 _Participant_ | | | 59 _Participant_ | | |
| Observations | 11700 | | | 11800 | | |
| Marginal R^2^ / Conditional R^2^ | 0.069 / 0.608 | | | 0.117 / 0.829 | | |

For outcome binding, we found a very small main effect of time ß=16.34, *95% CI=*[10.89 – 21.79], *p*<.001, *d*=0.108, no main effect of psychopathy (*p*=.439), and a small to medium sized interaction effect of time*psychopathy ß=47.59*95% CI=*[42.38 – 52.79], *p*<.001, *d*=0.331.

We found no main effect of sexual arousal (*p*=.714), but a very small sized interaction effect of sexual arousal*time ß=-10.94, *95% CI=*[-14.61 – -7.26], *p*<.001, *d*= -0.108. Further, we found no interaction of sexual arousal*psychopathy (*p*=.375), but a small sized interaction effect of time*sexual arousal*psychopathy, ß=-22.66, *95% CI=*[-26.29 – -19.02], *p*<.001, *d*=-0.225. Figure 7 shows that outcome binding is reduced at post measurement in the sexual arousal group in individuals high on psychopathy, whereas outcome binding does not differ in the neutral control group between pre and post measurement. For individuals low on psychopathy outcome binding does not differ between pre and post measurement in the sexual arousal group nor the control group.

We found no main effect of calm pleasure (*p*=.194), but a small sized interaction effect of calm pleasure and time ß= -57.90, *95% CI=*[-70.01 – -45.80], *p*<.001, *d*= -0.174. Further, we found no interaction of calm pleasure*psychopathy (*p*=.354), but a small to medium sized interaction effect of time*calm pleasure*psychopathy ß= -141.55, *95% CI=*[-153.50 – -129.61], *p*<.001, *d*= -0.429. Figure 7 shows that outcome binding in the neutral control group does not differ between pre and post measurement. However, outcome binding in the calm pleasure group seems to be reduced at post measurement in individuals high on psychopathy whereas outcome binding seems to be increased in the calm pleasure group in individuals low on psychopathy. The full model can be found in the Supplementary Table 12.

**Table 18**

*Model comparisons*

| Simple model | Model1 = action binding ~ group + time + (1\|Participant) | |
| --- | --- | --- |
| Action binding | Model2 = action binding ~ group* time + (1\| Participant) | |
|  | *χ2*=573.1 *p*<.001 | |
| Simple model | Model1 = outcome binding ~ group + time + (1\|Participant) | |
| Outcome binding | Model2 = outcome binding ~ group* time + (1\|Participant) | |
|  | *χ2*= 59.327 *p*<.001 | |
| Arousal model  Action binding | Model1 = action binding ~ group *time + blink rates + pupil dilation + arousal rating before each task + arousal rating after each task + (1\|Participant) | |
|  | Model2 = action binding ~ group*time*pupil dilation + group*time*blink rates + group*time* arousal rating before each task + group*time*arousal rating before each task +(1\| Participant) | |
|  | *χ2*= 3094.4 *p*<.001 | |
| Arousal model  Outcome binding | Model1 =outcome binding ~ group *time + blink rates + pupil dilation + arousal rating before each task + arousal rating after each task + (1\|Participant) | |
|  | Model2 = outcome binding ~ group*time* pupil dilation + group*time*blink rates + group*time* arousal rating before each task + group*time*arousal rating before each task +(1\| Participant) | |
|  | *χ2*= 4346.2 *p*<.001 | |
| Psychopathy model | Model1=action binding ~ group* time +Psychopathy+(1\|Participant) | |
| Action binding | Model2=action binding ~ group*time* Psychopathy+(1\|Participant) | |
|  | *χ2*= 485.44 *p*<.001 | |
| Psychopathy model | Model1=outcome binding ~ group* time + Psychopathy+(1\|Participant) |  |
| Outcome binding | Model2=outcome binding ~ group*time*Psychopathy +(1\|Participant) |  |
|  | *χ2*= 725.35 *p*<.001 | |
|  |  | |

## COVID

As our study took place close to the Covid-19 developments shortening the time frame to run participants, testing appointments took place after 5 pm (these participants did not have higher blinks rates than other participants) and contacts (not tracked) were not an exclusion criterion in contrast to most studies using eye blink rates.

1. **Methods and Results for Additional Hypotheses in Pre-registration**

***Questionnaires***

**Substance Use History**

Questions for frequency of substance use were extracted from the validated questionnaire of the European School Survey Project on Alcohol and Other Drugs (Kraus et al., 2016) as follows: On how many occasions (if any) have you used [insert substance]? The answer is split in two scales with seven possible categories (0, 1-2, 3-5, 6-9, 10-19, 20-39, 40 and more) for (a) in your lifetime, (b) during the last 12 months. Focus of substance were cannabis, amphetamine, methamphetamine, tranquilizers or sedatives, ecstasy, cocaine, LSD, ketamine, psilocybin, heroin, GHB or GBL. Sum scores for the number of different substances consumed in lifetime and during the last 12 months were calculated.

**Narcissism**

Vulnerable narcissism was measured with the Pathological Narcissism Inventory (PNI) (Pincus et al., 2009). The full version contains 52 items on a scale ranging from 1 (not at all like me) to 6 (very much like me). Evidence for validity and reliability is given (Cronbach’s Alpha *r* = 0.77) (Jakšić et al., 2014); Cronbach’s α in this sample was *r* = .91. The PNI was proposed as a measurement for both subtypes, narcissistic grandiosity (entitlement rage, exploitativeness, grandiose fantasy, self-sacrificing self-enhancement) and narcissistic vulnerability (contingent self-esteem, hiding the self, devaluing). Since factor analysis indicated that only one subscale (exploitativeness) represents the grandiose subtype (Miller et al., 2011), we used it to assess the vulnerable type only (40 Items) excluding the subscale exploitativeness and grandiose fantasies (loads on both factors alike).

Grandiose narcissism was assessed with the NPI-16, a short version of the NPI 40‐item self‐report assessment of trait narcissism (Raskin & Terry, 1988). The original NPI has four factors: Leadership/Authority, Superiority/Arrogance, Self-Absorption/Self-Admiration, and Entitlement/Exploitation. Reliability and validity have been confirmed in general (Kubarych et al., 2004; Raskin & Terry, 1988) and in this sample Cronbach’s α was *r* = .74. However, in accordance with (Miller et al., 2011) we only used 16 items loading on the two factors Leadership/Authority and Exhibitionism/Entitlement in this study. Factor analyses (Corry et al., 2008; Kubarych et al., 2004) showed a better replicability for these factors than other factor structures based on the NPI.

**Trait Anxiety**

The State‐Trait Anxiety Inventory (STAI) is an established, reliable, and brief self‐report scale for assessing state and trait anxiety in research and clinical practice (Spielberger, 1983). In this study, only the 20‐item trait anxiety scale was used to measure individual differences in anxiety proneness as a personality trait. Responses to items required subjects to indicate how they generally feel by reporting how often they have experienced anxiety‐related feelings and cognitions on a four‐point scale with almost never, sometimes, often and almost always. Cronbach’s α in this sample was *r* = .93.

**Personality characteristics**

Six one-way ANOVAs with between subject factor “group” and within subject factor “participant” for substance use history (lifetime), grandiose narcissism, vulnerable narcissism, psychopathy and trait anxiety confirmed, that the three groups only differ in grandiose narcissism but not in substance use history (lifetime), Vulnerable Narcissism and trait anxiety (Table 10). Grandiose narcissism was therefore not in included in analyses.

**Table 19**

*Mean, SD and group comparison for age, psychopathy score and trait anxiety score by group.*

| Variable | Group | | | Group comparison | |
| --- | --- | --- | --- | --- | --- |
|  | Sexual arousal | calm pleasure | Neutral control | *F*(2,56) | *p* |
| Substance Use History | 14.90(3.59) | 13.89(3.56) | 16.10(6.08) | 1.110 | .337 |
| Grandiose Narcissism | 3.48(2.79) | 2.33(2.22) | 4.70(3.36) | 3.285 | <.05 |
| Vulnerable Narcissism | 137.33(27.93) | 123.28(25.65) | 125.45(21.92) | 1.793 | .176 |
| Trait Anxiety | 46.67(11.32) | 45.89(8.36) | 43.80(9.40) | 0.447 | .642 |

**Figure 11**

*Difference scores for binding (post – pre) by binding component for personality traits and substance use history. Above 0 = increase in binding from pre- to post-measurement, below 0 = decrease in binding from pre- to post-measurement.*


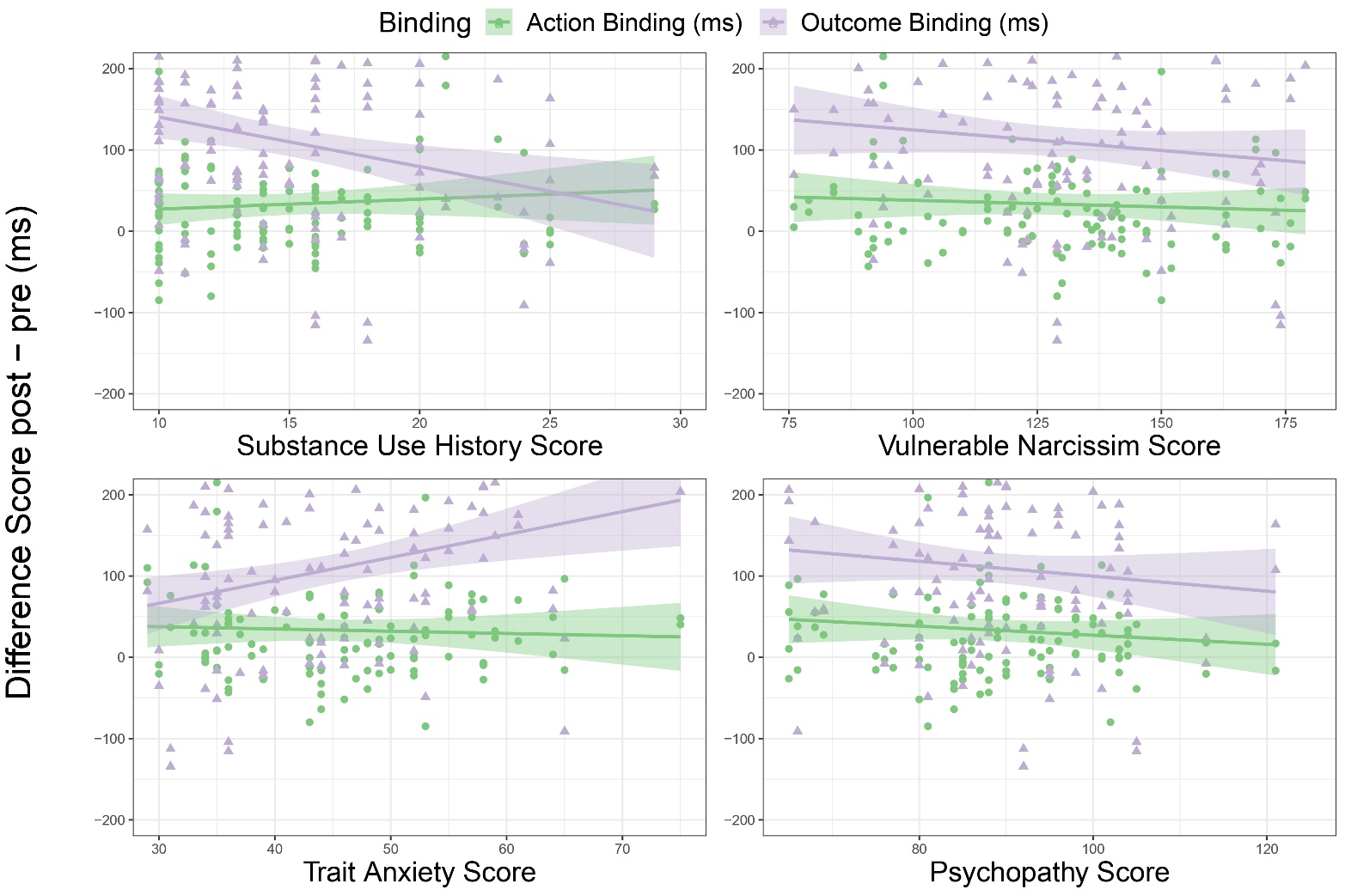


**Results**

**Substance Use History**

**Table 20**

*Linear mixed models for substance use history with two different sets of orthogonal contrasts.*

*Dependent variable = action binding or outcome binding,*

*fixed term structure = time, group, substance use history, time by group, time by substance use history, group by substance use history, time by group by substance use history*

*random term structure = participants*

1. Neutral control vs. sexual arousal

*Contrast 1: Neutral control = -1, calm pleasant = 2, sexual arousal = -1*

*Contrast 2: Neutral control = 1, calm pleasant = 0, sexual arousal = -1*

|  | **Action binding** | | | **Outcome binding** | | |
| --- | --- | --- | --- | --- | --- | --- |
| *Predictors* | *Estimates* | *CI* | *p* | *Estimates* | *CI* | *p* |
| (Intercept) | 31.91 | 21.17 – 42.66 | **<.001** | 111.53 | 89.69 – 133.37 | **<.001** |
| Time (pre vs. post) | -0.27 | -0.94 – 0.39 | 0.422 | 0.12 | -0.64 – 0.87 | 0.765 |
| Calm pleasure vs. sexual arousal and neutral control | 6.99 | -0.87 – 14.86 | 0.082 | 16.66 | 0.67 – 32.65 | **0.041** |
| Sexual arousal (neutral control vs. sexual arousal) | -0.94 | -13.61 – 11.74 | 0.885 | 6.48 | -19.29 – 32.24 | 0.622 |
| Substance use history | 10.12 | -2.04 – 22.28 | 0.103 | -26.08 | -50.80 – -1.36 | **0.039** |
| Time (pre vs. post) * Calm pleasure vs. sexual arousal and neutral control | -0.02 | -0.51 – 0.48 | 0.946 | 0.81 | 0.25 – 1.36 | **0.004** |
| Time (pre vs. post) * Sexual arousal (neutral control vs. sexual arousal) | 8.47 | 7.69 – 9.24 | **<.001** | 4.60 | 3.71 – 5.50 | **<.001** |
| Time (pre vs. post) * Substance use history | 4.31 | 3.56 – 5.06 | **<.001** | 7.14 | 6.28 – 8.00 | **<.001** |
| Calm pleasure vs. sexual arousal and neutral control * Substance use history | 9.49 | 0.07 – 18.91 | **0.048** | 2.66 | -16.49 – 21.80 | 0.786 |
| Sexual arousal (neutral control vs. sexual arousal) * Substance use history | 4.91 | -8.42 – 18.23 | 0.470 | 6.81 | -20.28 – 33.90 | 0.622 |
| Time (pre vs. post) * Calm pleasure vs. sexual arousal and neutral control) * Substance use history | -4.47 | -5.05 – -3.89 | **<.001** | 2.27 | 1.60 – 2.93 | **<.001** |
| Time (pre vs. post) * Sexual arousal (neutral control vs. sexual arousal) * Substance use history | 2.05 | 1.24 – 2.87 | **<.001** | -14.82 | -15.76 – -13.88 | **<.001** |
| **Random Effects** | | | | | | |
| σ^2^ | 1270.47 | | | 1675.50 | | |
| τ_00_ | 1676.42 _Participant_ | | | 6945.42 _Participant_ | | |
| ICC | 0.57 | | | 0.81 | | |
| N | 59 _Participant_ | | | 59 _Participant_ | | |
| Observations | 11700 | | | 11800 | | |
| Marginal R^2^ / Conditional R^2^ | 0.109 / 0.616 | | | - 1. 0.834 | | |

1. Neutral control vs. calm pleasure

*Contrast 1: Neutral control = -1, calm pleasant = -1, sexual arousal = 2*

*Contrast 2: Neutral control = 1, calm pleasant = -1, sexual arousal = 0*

|  | **Action binding** | | | **Outcome binding** | | |
| --- | --- | --- | --- | --- | --- | --- |
| *Predictors* | *Estimates* | *CI* | *p* | *Estimates* | *CI* | *p* |
| (Intercept) | 31.91 | 21.17 – 42.66 | **<.001** | 111.53 | 89.69 – 133.37 | **<.001** |
| Time (pre vs. post) | -0.27 | -0.94 – 0.39 | 0.422 | 0.12 | -0.64 – 0.87 | 0.765 |
| Sexual arousal vs. calm pleasure and neutral control | -3.03 | -10.41 – 4.36 | 0.422 | -11.57 | -26.58 – 3.45 | 0.131 |
| Calm pleasure (neutral control vs. calm pleasure) | -10.96 | -24.47 – 2.56 | 0.112 | -21.75 | -49.22 – 5.73 | 0.121 |
| Substance use history | 10.12 | -2.04 – 22.28 | 0.103 | -26.08 | -50.80 – -1.36 | **0.039** |
| Time (pre vs. post) * Sexual arousal vs. calm pleasure and neutral control | -4.22 | -4.68 – -3.77 | **<.001** | -2.70 | -3.22 – -2.18 | **<.001** |
| Time (pre vs. post) * Calm pleasure (neutral control vs. calm pleasure) | 4.26 | 3.42 – 5.10 | **<.001** | 1.09 | 0.14 – 2.05 | **0.025** |
| Time (pre vs. post) * Substance use history | 4.31 | 3.56 – 5.06 | **<.001** | 7.14 | 6.28 – 8.00 | **<.001** |
| Sexual arousal vs. calm pleasure and neutral control * Substance use history | -7.20 | -16.16 – 1.76 | 0.115 | -4.73 | -22.95 – 13.48 | 0.610 |
| Calm pleasure (neutral control vs. calm pleasure) * Substance use history | -11.78 | -26.02 – 2.46 | 0.105 | -0.58 | -29.53 – 28.38 | 0.969 |
| Time (pre vs. post) * Sexual arousal vs. calm pleasure and neutral control * Substance use history | 1.21 | 0.66 – 1.76 | **<.001** | 6.28 | 5.65 – 6.91 | **<.001** |
| Time (pre vs. post) * Calm pleasure (neutral control vs. calm pleasure)) * Substance use history | 7.73 | 6.86 – 8.61 | **<.001** | -10.81 | -11.81 – -9.80 | **<.001** |
| **Random Effects** | | | | | | |
| σ^2^ | 1270.47 | | | 1675.50 | | |
| τ_00_ | 1676.42 _Participant_ | | | 6945.42 _Participant_ | | |
| ICC | 0.57 | | | 0.81 | | |
| N | 59 _Participant_ | | | 59 _Participant_ | | |
| Observations | 11700 | | | 11800 | | |
| Marginal R^2^ / Conditional R^2^ | 0.109 / 0.616 | | | 0.148 / 0.834 | | |

**Figure 12**

*Change in action and outcome binding between pre- and post-induction task by group (difference score, post-pre) for substance use history. Above 0 = increase in binding from pre- to post-measurement, below 0 = decrease in binding from pre- to post-measurement.*


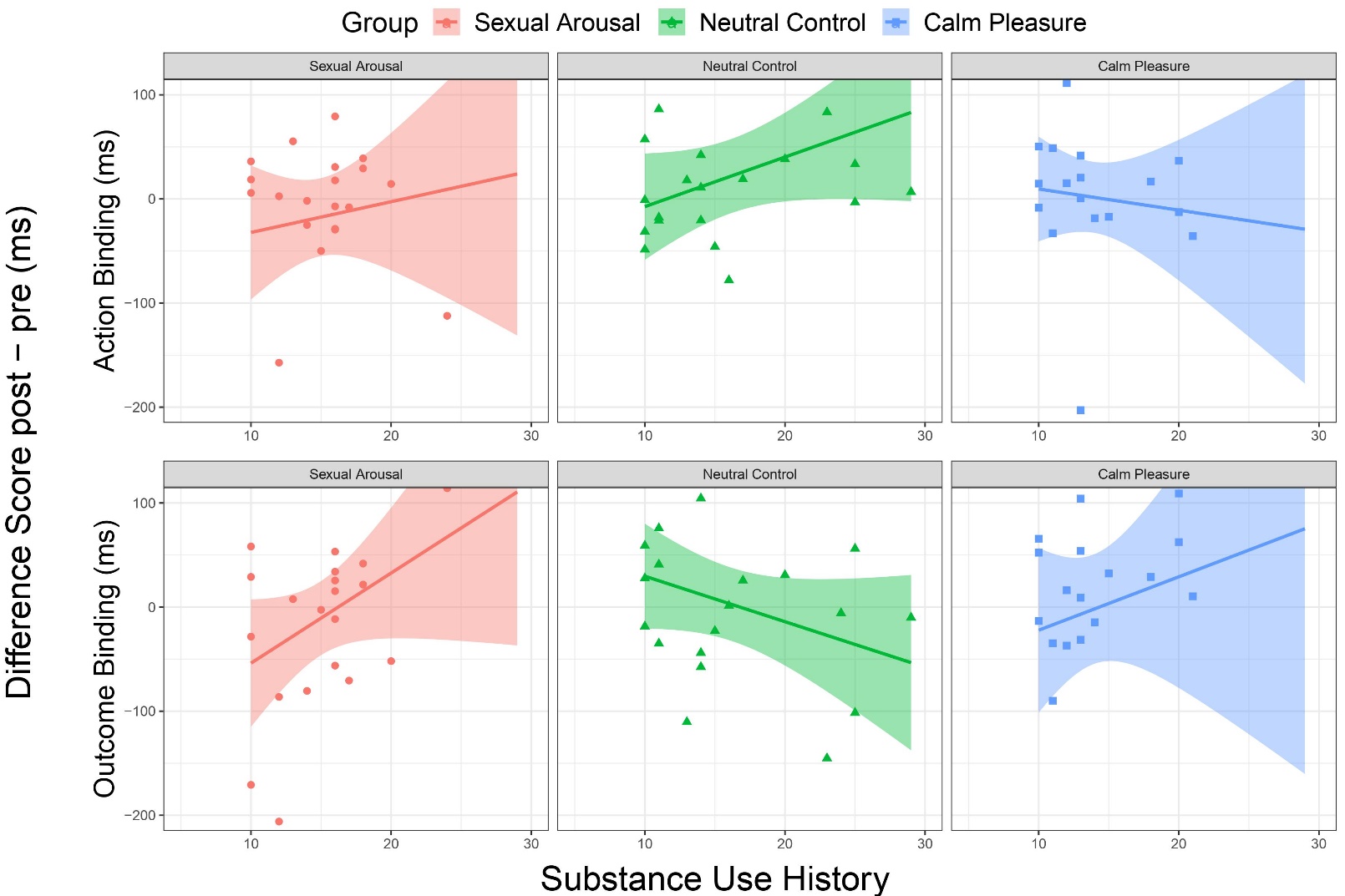


**Vulnerable Narcissism**

**Table 21**

*Simple linear mixed models for vulnerable narcissism with two different sets of orthogonal contrasts.*

*Dependent variable = action binding or outcome binding,*

*fixed term structure = time, group, vulnerable narcissism, time by group, time by vulnerable narcissism, group by vulnerable narcissism, time by group by vulnerable narcissism*

*random term structure = participants*

1. Neutral control vs. sexual arousal

*Contrast 1: Neutral control = -1, calm pleasant = 2, sexual arousal = -1*

*Contrast 2: Neutral control = 1, calm pleasant = 0, sexual arousal = -1*

|  | **Action binding** | | | **Outcome binding** | | |
| --- | --- | --- | --- | --- | --- | --- |
| *Predictors* | *Estimates* | *CI* | *p* | *Estimates* | *CI* | *p* |
| (Intercept) | 30.55 | 19.28 – 41.82 | **<.001** | 114.11 | 91.68 – 136.55 | **<.001** |
| Time (pre vs. post) | 0.90 | 0.22 – 1.59 | **0.010** | -1.77 | -2.56 – -0.98 | **<.001** |
| Calm pleasure vs. sexual arousal and neutral control | 4.09 | -4.05 – 12.22 | 0.325 | 16.76 | 0.57 – 32.95 | **0.042** |
| Sexual arousal (neutral control vs. sexual arousal) | -1.48 | -14.99 – 12.03 | 0.830 | 0.89 | -26.00 – 27.78 | 0.948 |
| Vulnerable Narcissism | -2.96 | -14.52 – 8.60 | 0.615 | -5.61 | -28.62 – 17.40 | 0.633 |
| Time (pre vs. post) * Calm pleasure vs. sexual arousal and neutral control | -0.19 | -0.69 – 0.32 | 0.468 | -0.43 | -1.00 – 0.14 | 0.142 |
| Time (pre vs. post) * Sexual arousal (neutral control vs. sexual arousal) | 8.98 | 8.17 – 9.80 | **<.001** | 3.79 | 2.85 – 4.74 | **<.001** |
| Time (pre vs. post) * Vulnerable Narcissism | -3.21 | -3.90 – -2.51 | **<.001** | -0.56 | -1.37 – 0.25 | 0.176 |
| Calm pleasure vs. sexual arousal and neutral control * Vulnerable Narcissism | 1.31 | -6.90 – 9.53 | 0.754 | -2.24 | -18.59 – 14.11 | 0.788 |
| Sexual arousal (neutral control vs. sexual arousal) * Vulnerable Narcissism | 1.81 | -12.28 – 15.90 | 0.801 | 19.05 | -8.99 – 47.09 | 0.183 |
| Time (pre vs. post) * Calm pleasure vs. sexual arousal and neutral control) * Vulnerable Narcissism | 0.98 | 0.48 – 1.47 | **<.001** | -3.06 | -3.64 – -2.48 | **<.001** |
| Time (pre vs. post) * Sexual arousal (neutral control vs. sexual arousal) * Vulnerable Narcissism | -1.22 | -2.07 – -0.37 | **0.005** | 4.23 | 3.24 – 5.22 | **<.001** |
| **Random Effects** | | | | | | |
| σ^2^ | 1341.15 | | | 1813.81 | | |
| τ_00_ | 1830.53 _Participant_ | | | 7269.82 _Participant_ | | |
| ICC | 0.58 | | | 0.80 | | |
| N | 59 _Participant_ | | | 59 _Participant_ | | |
| Observations | 11700 | | | 11800 | | |
| Marginal R^2^ / Conditional R^2^ | 0.040 / 0.594 | | | - 1. 0.821 | | |

1. Neutral control vs. calm pleasure

*Contrast 1: Neutral control = -1, calm pleasant = -1, sexual arousal = 2*

*Contrast 2: Neutral control = 1, calm pleasant = -1, sexual arousal = 0*

|  | **Action binding** | | | **Outcome binding** | | |
| --- | --- | --- | --- | --- | --- | --- |
| *Predictors* | *Estimates* | *CI* | *p* | *Estimates* | *CI* | *p* |
| (Intercept) | 30.55 | 19.28 – 41.82 | **<.001** | 114.11 | 91.68 – 136.55 | **<.001** |
| Time (pre vs. post) | 0.90 | 0.22 – 1.59 | **0.010** | -1.77 | -2.56 – -0.98 | **<.001** |
| Sexual arousal vs. calm pleasure and neutral control | -1.30 | -9.20 – 6.59 | 0.746 | -8.83 | -24.55 – 6.90 | 0.271 |
| Calm pleasure (neutral control vs. calm pleasure) | -6.87 | -20.80 – 7.05 | 0.333 | -24.69 | -52.41 – 3.03 | 0.081 |
| Vulnerable Narcissism | -2.96 | -14.52 – 8.60 | 0.615 | -5.61 | -28.62 – 17.40 | 0.633 |
| Time (pre vs. post) * Sexual arousal vs. calm pleasure and neutral control | -4.40 | -4.88 – -3.92 | **<.001** | -1.68 | -2.24 – -1.13 | **<.001** |
| Time (pre vs. post) * Calm pleasure (neutral control vs. calm pleasure) | 4.77 | 3.91 – 5.62 | **<.001** | 2.54 | 1.56 – 3.52 | **<.001** |
| Time (pre vs. post) * Vulnerable Narcissism | -3.21 | -3.90 – -2.51 | **<.001** | -0.56 | -1.37 – 0.25 | 0.176 |
| Sexual arousal vs. calm pleasure and neutral control * Vulnerable Narcissism | -1.56 | -9.17 – 6.04 | 0.687 | -8.40 | -23.54 – 6.74 | 0.277 |
| Calm pleasure (neutral control vs. calm pleasure) * Vulnerable Narcissism | -1.07 | -16.14 – 14.01 | 0.890 | 12.89 | -17.13 – 42.90 | 0.400 |
| Time (pre vs. post) * Sexual arousal vs. calm pleasure and neutral control * Vulnerable Narcissism | 0.12 | -0.34 – 0.58 | 0.606 | -0.58 | -1.12 – -0.05 | **0.032** |
| Time (pre vs. post) * Calm pleasure (neutral control vs. calm pleasure) * Vulnerable Narcissism | -2.07 | -2.98 – -1.16 | **<.001** | 6.70 | 5.64 – 7.76 | **<.001** |
| **Random Effects** | | | | | | |
| σ^2^ | 1341.15 | | | 1813.81 | | |
| τ_00_ | 1830.53 _Participant_ | | | 7269.82 _Participant_ | | |
| ICC | 0.58 | | | 0.80 | | |
| N | 59 _Participant_ | | | 59 _Participant_ | | |
| Observations | 11700 | | | 11800 | | |
| Marginal R^2^ / Conditional R^2^ | 0.040 / 0.594 | | | 0.103 / 0.821 | | |

**Figure 13**

*Change in action and outcome binding between pre- and post-induction task by group (difference score, post-pre) for Vulnerable Narcissism. Above 0 = increase in binding from pre- to post-measurement, below 0 = decrease in binding from pre- to post-measurement.*


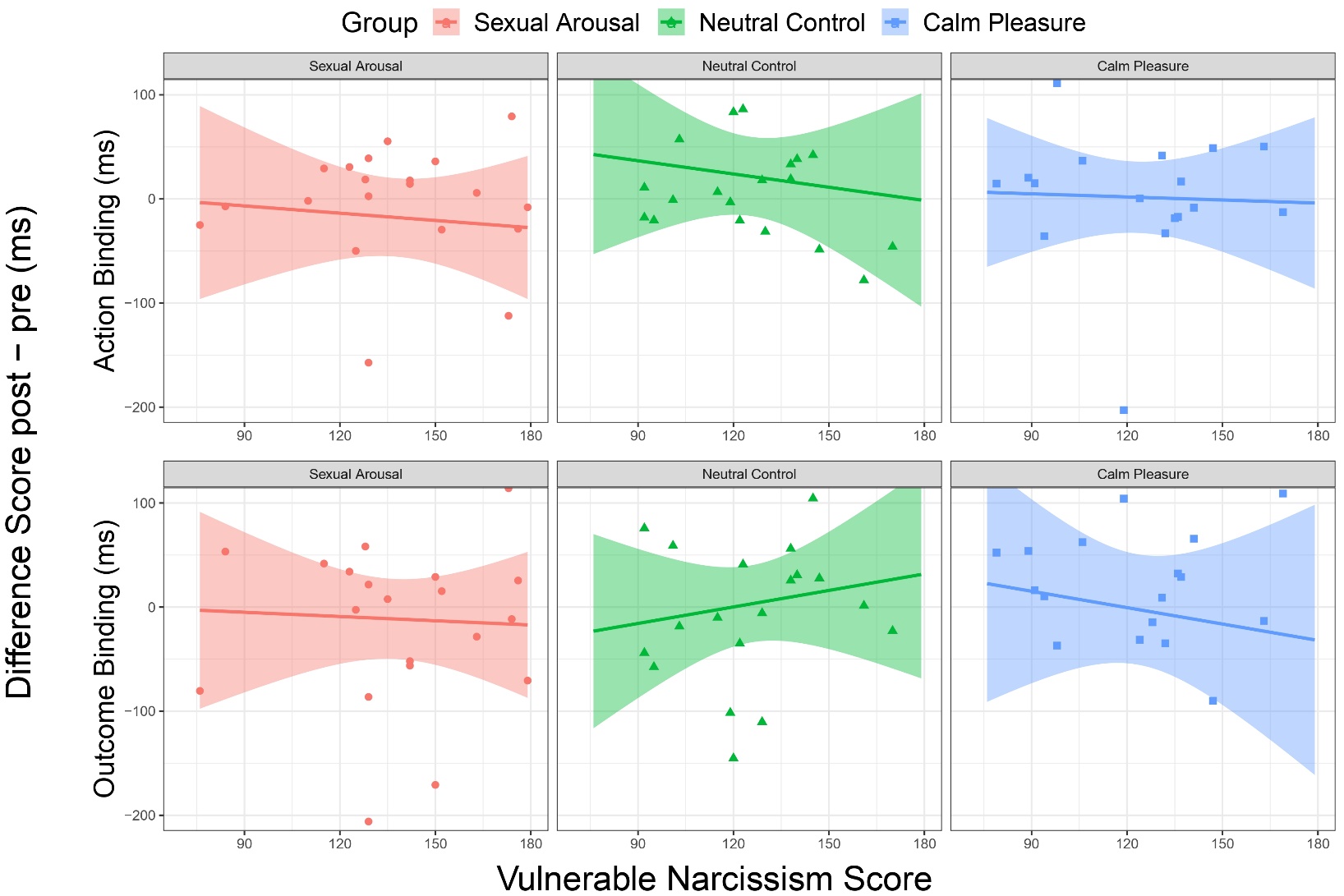


**Trait Anxiety**

**Table 22**

*Simple linear mixed models for trait anxiety with two different sets of orthogonal contrasts.*

*Dependent variable = action binding or outcome binding,*

*fixed term structure = time, group, trait anxiety, time by group, time by trait anxiety, group by trait anxiety, time by group by trait anxiety*

*random term structure = participants*

1. Neutral control vs. sexual arousal

*Contrast 1: Neutral control = -1, calm pleasant = 2, sexual arousal = -1*

*Contrast 2: Neutral control = 1, calm pleasant = 0, sexual arousal = -1*

|  | **Action binding** | | | **Outcome binding** | | |
| --- | --- | --- | --- | --- | --- | --- |
| *Predictors* | *Estimates* | *CI* | *p* | *Estimates* | *CI* | *p* |
| (Intercept) | 29.64 | 18.60 – 40.69 | **<.001** | 111.84 | 90.57 – 133.12 | **<.001** |
| Time (pre vs. post) | 0.77 | 0.12 – 1.43 | **0.021** | -1.47 | -2.22 – -0.71 | **<.001** |
| Calm pleasure vs. sexual arousal and neutral control | 4.63 | -3.32 – 12.58 | 0.254 | 18.44 | 3.12 – 33.76 | **0.018** |
| Sexual arousal (neutral control vs. sexual arousal) | -0.52 | -13.79 – 12.75 | 0.939 | 7.59 | -17.98 – 33.15 | 0.561 |
| Trait Anxiety | -1.91 | -13.48 – 9.66 | 0.746 | 27.70 | 5.41 – 49.99 | **0.015** |
| Time (pre vs. post) * Calm pleasure vs. sexual arousal and neutral control | 0.11 | -0.37 – 0.58 | 0.662 | -0.30 | -0.84 – 0.24 | 0.280 |
| Time (pre vs. post) * Sexual arousal (neutral control vs. sexual arousal) | 8.25 | 7.48 – 9.03 | **<.001** | 4.49 | 3.59 – 5.40 | **<.001** |
| Time (pre vs. post) * Trait Anxiety | -7.86 | -8.54 – -7.17 | **<.001** | 10.59 | 9.81 – 11.38 | **<.001** |
| Calm pleasure vs. sexual arousal and neutral control * TA | -1.14 | -10.06 – 7.78 | 0.803 | -0.37 | -17.57 – 16.82 | 0.966 |
| Sexual arousal (neutral control vs. sexual arousal) * Trait Anxiety | -4.13 | -16.89 – 8.63 | 0.526 | 1.11 | -23.47 – 25.69 | 0.929 |
| Time (pre vs. post) * Calm pleasure vs. sexual arousal and neutral control) * Trait Anxiety | 2.94 | 2.42 – 3.47 | **<.001** | 5.38 | 4.78 – 5.99 | **<.001** |
| Time (pre vs. post) * Sexual arousal (neutral control vs. sexual arousal)) * Trait Anxiety | -0.48 | -1.23 – 0.27 | 0.211 | 5.55 | 4.68 – 6.42 | **<.001** |
| **Random Effects** | | | | | | |
| σ^2^ | 1262.17 | | | 1704.52 | | |
| τ_00_ | 1830.31 _Participant_ | | | 6810.67 _Participant_ | | |
| ICC | 0.59 | | | 0.80 | | |
| N | 59 _Participant_ | | | 59 _Participant_ | | |
| Observations | 11700 | | | 11800 | | |
| Marginal R^2^ / Conditional R^2^ | 0.064 / 0.618 | | | - 1. 0.832 | | |

1. control vs. calm pleasure

*Contrast 1: control = -1, calm pleasant = -1, sexual arousal = 2*

*Contrast 2: control = 1, calm pleasant = -1, sexual arousal = 0*

|  | **Action binding** | | | **Outcome binding** | | |
| --- | --- | --- | --- | --- | --- | --- |
| *Predictors* | *Estimates* | *CI* | *p* | *Estimates* | *CI* | *p* |
| (Intercept) | 29.64 | 18.60 – 40.69 | **<.001** | 111.84 | 90.57 – 133.12 | **<.001** |
| Time (pre vs. post) | 0.77 | 0.12 – 1.43 | **0.021** | -1.47 | -2.22 – -0.71 | **<.001** |
| Sexual arousal vs. calm pleasure and neutral control | -2.05 | -9.72 – 5.61 | 0.599 | -13.01 | -27.79 – 1.76 | 0.084 |
| Calm pleasure (neutral control vs. calm pleasure) | -7.20 | -20.96 – 6.56 | 0.305 | -23.87 | -50.38 – 2.64 | 0.078 |
| Trait Anxiety | -1.91 | -13.48 – 9.66 | 0.746 | 27.70 | 5.41 – 49.99 | **0.015** |
| Time (pre vs. post) * Sexual arousal vs. calm pleasure and neutral control | -4.18 | -4.63 – -3.73 | **<.001** | -2.10 | -2.62 – -1.58 | **<.001** |
| Time (pre vs. post) * Calm pleasure (neutral control vs. calm pleasure) | 3.97 | 3.15 – 4.79 | **<.001** | 2.70 | 1.76 – 3.63 | **<.001** |
| Time (pre vs. post) * Trait Anxiety | -7.86 | -8.54 – -7.17 | **<.001** | 10.59 | 9.81 – 11.38 | **<.001** |
| Sexual arousal vs. calm pleasure and neutral control * Trait Anxiety | 2.63 | -4.78 – 10.05 | 0.486 | -0.37 | -14.65 – 13.91 | 0.960 |
| Calm pleasure (neutral control vs. calm pleasure) * Trait Anxiety | -0.36 | -15.74 – 15.03 | 0.964 | 1.12 | -28.53 – 30.77 | 0.941 |
| Time (pre vs. post) * Sexual arousal vs. calm pleasure and neutral control * Trait Anxiety | -1.23 | -1.67 – -0.80 | **<.001** | -5.47 | -5.97 – -4.96 | **<.001** |
| Time (pre vs. post) * Calm pleasure (neutral control vs. calm pleasure)) * Trait Anxiety | -4.66 | -5.57 – -3.75 | **<.001** | -5.30 | -6.35 – -4.26 | **<.001** |
| **Random Effects** | | | | | | |
| σ^2^ | 1262.17 | | | 1704.52 | | |
| τ_00_ | 1830.31 _Participant_ | | | 6810.67 _Participant_ | | |
| ICC | 0.59 | | | 0.80 | | |
| N | 59 _Participant_ | | | 59 _Participant_ | | |
| Observations | 11700 | | | 11800 | | |
| Marginal R^2^ / Conditional R^2^ | 0.064 / 0.618 | | | 0.159 / 0.832 | | |

**Figure 14**

*Change in action and outcome binding between pre- and post-induction task by group (difference score, post-pre) for trait anxiety. Above 0 = increase in binding from pre- to post-measurement, below 0 = decrease in binding from pre- to post-measurement.*


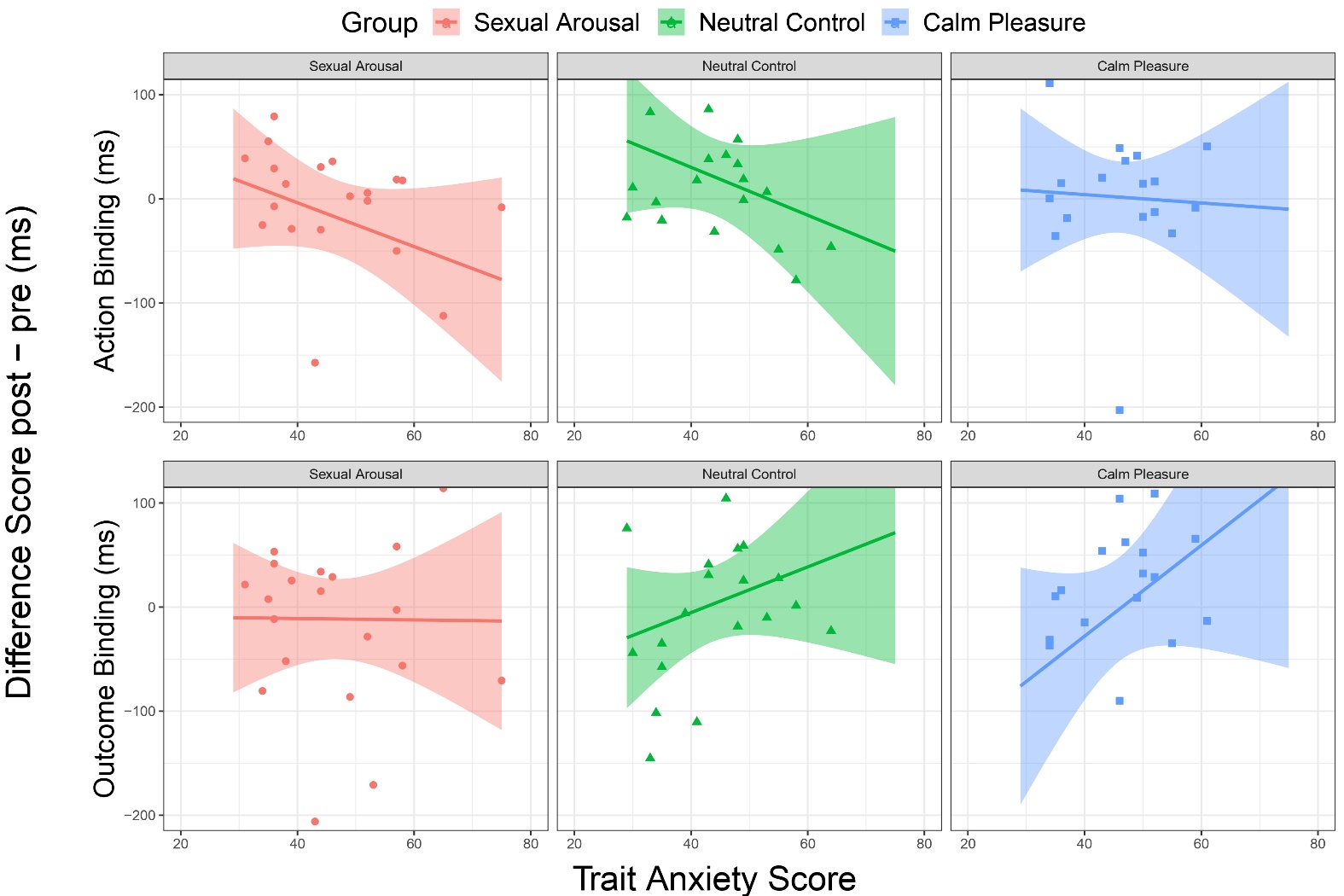


Publication bibliography

Corry, Nida; Merritt, Rebecca Davis; Mrug, Sylvie; Pamp, Barbara (2008): The factor structure of the Narcissistic Personality Inventory. In *Journal of personality assessment* 90 (6), pp. 593–600. DOI: 10.1080/00223890802388590.

Gabert-Quillen, Crystal A.; Bartolini, Ellen E.; Abravanel, Benjamin T.; Sanislow, Charles A. (2015): Ratings for emotion film clips. In *Behavior research methods* 47 (3), pp. 773–787. DOI: 10.3758/s13428-014-0500-0.

Jakšić, Nenad; Milas, Goran; Ivezić, Ena; Wertag, Anja; Jokić-Begić, Nataša; Pincus, Aaron L. (2014): The Pathological Narcissism Inventory (PNI) in Transitional Post-War Croatia: Psychometric and Cultural Considerations. In *J Psychopathol Behav Assess* 36 (4), pp. 640–652. DOI: 10.1007/s10862-014-9425-2.

Kraus, Ludwig; Leifman, Håkan; Vicente, Julian; Guttormsson, Ulf; Molinaro, Sabrina; Arpa, Sharon (2016): ESPAD Report 2015. Results from the European School Survey Project on Alcohol and Other Drugs. Luxembourg: Publications Office of the European Union (ESPAD Report, 2015).

Kubarych, Thomas S.; Deary, Ian J.; Austin, Elizabeth J. (2004): The Narcissistic Personality Inventory: factor structure in a non-clinical sample. In *Personality and Individual Differences* 36 (4), pp. 857–872. DOI: 10.1016/S0191-8869(03)00158-2.

Miller, J. D.; Hoffman, Brian J.; Gaughan, Eric T.; Gentile, Brittany; Maples, Jessica; Keith Campbell, W. (2011): Grandiose and vulnerable narcissism: a nomological network analysis. In *Journal of personality* 79 (5), pp. 1013–1042. DOI: 10.1111/j.1467-6494.2010.00711.x.

Pincus, Aaron L.; Ansell, Emily B.; Pimentel, Claudia A.; Cain, Nicole M.; Wright, Aidan G. C.; Levy, Kenneth N. (2009): Initial construction and validation of the Pathological Narcissism Inventory. In *Psychological Assessment: A Journal of Consulting and Clinical Psychology* 21 (3), pp. 365–379. DOI: 10.1037/a0016530.

Ramzan, Naeem; Palke, Sebastian; Cuntz, Thomas; Gibson, Ryan; Amira, Abbes (2016): Emotion Recognition by Physiological Signals. In *Electronic Imaging* 2016 (16), pp. 1–6. DOI: 10.2352/ISSN.2470-1173.2016.16.HVEI-129.

Raskin, R. N.; Terry, Howard (1988): A principal-components analysis of the Narcissistic Personality Inventory and further evidence of its construct validity. In *Journal of Personality and Social Psychology* 54 (5), pp. 890–902. DOI: 10.1037/0022-3514.54.5.890.

Spielberger, C. D. (1983): State-trait anxiety inventory for adults.
